# Quantitative Laser Biospeckle Method for the Evaluation of the Activity of *Trypanosoma cruzi* Using VDRL Plates and Digital Analysis

**DOI:** 10.1371/journal.pntd.0005169

**Published:** 2016-12-05

**Authors:** Hilda Cristina Grassi, Lisbette C. García, María Lorena Lobo-Sulbarán, Ana Velásquez, Francisco A. Andrades-Grassi, Humberto Cabrera, Jesús E. Andrades-Grassi, Efrén D. J. Andrades

**Affiliations:** 1 Facultad de Farmacia y Bionálisis, Universidad de Los Andes, Mérida, Venezuela; 2 Cátedra de Farmacología y Terapéutica, Departamento de Biopatología, Facultad de Odontología, Universidad de Los Andes, Mérida, Venezuela; 3 Centro Multidisciplinario de Ciencias, Instituto Venezolano de Investigaciones Científicas, Mérida, Venezuela; 4 Ogangi Corporation, Miami, Florida, United States of America; 5 SPIE-ICTP Anchor Research in Optics Program Laboratory, International Centre for Theoretical Physics, Trieste, Italy; 6 Facultad de Ciencias Forestales y Ambientales, Departamento de Ordenación de Cuencas, Universidad de Los Andes Mérida, Venezuela; New York University School of Medicine, UNITED STATES

## Abstract

In this paper we report a quantitative laser Biospeckle method using VDRL plates to monitor the activity of *Trypanosoma cruzi* and the calibration conditions including three image processing algorithms and three programs (ImageJ and two programs designed in this work). Benznidazole was used as a test drug. Variable volume (constant density) and variable density (constant volume) were used for the quantitative evaluation of parasite activity in calibrated wells of the VDRL plate. The desiccation process within the well was monitored as a function of volume and of the activity of the Biospeckle pattern of the parasites as well as the quantitative effect of the surface parasite quantity (proportion of the object’s plane). A statistical analysis was performed with ANOVA, Tukey *post hoc* and Descriptive Statistics using R and R Commander. Conditions of volume (100μl) and parasite density (2-4x10^4^ parasites/well, in exponential growth phase), assay time (up to 204min), frame number (11 frames), algorithm and program (RCommander/SAGA) for image processing were selected to test the effect of variable concentrations of benznidazole (0.0195 to 20μg/mL / 0.075 to 76.8μM) at various times (1, 61, 128 and 204min) on the activity of the Biospeckle pattern. The flat wells of the VDRL plate were found to be suitable for the quantitative calibration of the activity of *Trypanosoma cruzi* using the appropriate algorithm and program. Under these conditions, benznidazole produces at 1min an instantaneous effect on the activity of the Biospeckle pattern of *T*. *cruzi*, which remains with a similar profile up to 1 hour. A second effect which is dependent on concentrations above 1.25μg/mL and is statistically different from the effect at lower concentrations causes a decrease in the activity of the Biospeckle pattern. This effect is better detected after 1 hour of drug action. This behavior may be explained by an instantaneous effect on a membrane protein of *Trypanosoma cruzi* that could mediate the translocation of benznidazole. At longer times the effect may possibly be explained by the required transformation of the pro-drug into the active drug.

## Author Summary

Biospeckle refers to a pattern that occurs when a laser beam illuminates a dynamic surface, such as a liquid that contains microorganisms. The movement or the roughness of its surface causes the wave fronts to interfere and produce a pattern of moving dots that resemble boiling water. This research describes the application of Biospeckle to *Trypanosoma cruzi*, the parasite that causes Chagas disease. The purpose was to observe the movement of the Biospeckle dots and to detect differences depending on the presence of the parasite, the quantity of the parasite and the conditions of the parasites when they are affected by a drug. We designed a method using VDRL plates where the sample has a relatively small volume and is flat shaped, a laser, a camera and a lens. The Biospeckle pattern is recorded in a video in a computer and shows the Biospeckle dots which move rapidly as the concentration of parasites increases and less rapidly as the concentration decreases or as the parasites are affected by a drug such as benznidazole. We designed algorithms which take the difference between successive frames and expressed them in a program in Java, in a script in R Commander and SAGA and in ImageJ. Thus we obtained a quantitative description of the movement of T. cruzi.

## Introduction

Chemotherapy is the most widely used method to control, prevent and treat parasitic infections. In order to understand the action of a drug on a parasite, *in vitro* assay techniques that test its activity, as well as the physical factors that affect the parasites are performed under the best conditions that allow the detection and quantification of the effect, avoiding or decreasing random factors and using fast, linear, sensitive, accurate, precise, inexpensive and reproducible methods [1].

The activity of most anti-parasitic molecules is based on their ability to permeate, to reach a specific receptor inside the parasite, to obtain delivery of effective drug concentrations in sufficient time to cause the therapeutic effect before it is degraded, transformed or eliminated [2]. Understanding the relationship of the anti-parasitic effect with concentration and time is crucial for this rational. Moreover, recently, efforts have focused on combination of drugs in order to aim at new therapies as a more effective alternative in the treatment of Chagas’ disease [3].

The design of *in vitro* assays must take into account that parasites are very sensitive to physical, chemical and biological factors such as handling, temperature, evaporation, osmolarity changes, nutritional factors, doubling time, etc. The assays that are based on viability require a low dose of the test drug and several hours and even days to develop [4]. Therefore, the use of methods that require a short time are useful in avoiding undesired factors such as degradation, biotransformation and other effects on the test drug. A fast assay could be useful for the detection of the first stages of a drug’s pharmacological activity. Image processing using new algorithms has already been proposed for *T*. *cruzi* detection and drug testing [1][5][6]. However, it is desirable to develop algorithms, programs and free and open source software, to quantify microorganisms, especially in the case of Neglected Tropical Diseases that are frequently addressed under limited conditions.

In the present experimental work, we describe a novel assay methodology for the *in vitro* testing of parasite activity using a VDRL plate. The methodology is based on laser dynamic speckle interferometry, called Biospeckle interferometry [7][8]. Different Biospeckle descriptors have been proposed and evaluated from the performance point of view [9] and others have explored the combination of spatial—temporal patterns for the analysis of the dynamics of slow-varying phenomena [10]. In the present work the Biospeckle technique is applied to several samples, following the time evolution of the activity of the Biospeckle patterns. Epimastigotes of *Trypanosoma cruzi* M/HOM/ VE/67 /EP-67 were used as the experimental model. In order to standardize the method we evaluated variable amounts of the parasite and benznidazole as a test drug with a combination of high concentration and short time, as well as different computational approaches for the processing of the laser Biospeckle interferometry images, using flat test wells of VDRL plates as a novel sample container for this technique. The VDRL plate was recommended as a specific and reproducible system by the World Health Organization in 1980 [11]. The design and dimensions of the VDRL flat test wells are adapted to optical microscopes and magnifiers and they are available in most health centers worldwide. Similarly, other authors have adapted flat-bottomed 96 well micro-plates to quantifying the effects of drugs, antibodies and gene modifications on parasite fitness and replication rates of the human malaria parasite *Plasmodium falciparum*, another agent of a tropical disease [12].

## Materials and Methods

### VDRL assay plate

VDRL Plates were used with the following supplier’s information: each plate of pressed glass has the dimensions 89x57x4.5mm, with 12 numbered cavities of approximately 1.5mm depth and 15mm external diameter with overflow grooves. In order to test reproducibility, the internal diameter of the cavities, 60 wells (5 plates) were measured under a calibrated magnifier, obtaining a Mean internal diameter of 12.7+/- 0.1 mm. With these dimensions and the approximate size of the parasite (length 20μm and width 5μm, mean 12.5μm), the object’s plane (surface disk containing the most exposed parasites) available for the Biospeckle technique has a mean height of 0.0125 mm, a mean volume of 1.66μL and contains approximately 670 parasites when the density is 4x10^5^ parasites per mL (4x10^4^ parasites per well). Unless otherwise stated, all the assays were performed with an initial volume of 100μL.

### Parasites and culture conditions

*Trypanosoma cruzi* strain M/HOM/ VE/67 /EP-67 was used. The parasites were grown in Liver Infusion Tryptose (LIT) medium with 5% Neonatal Bovine Serum [13] at 28°C. Just before performing each experiment, parasites were counted in a Neubauer chamber and used when cultures were at exponential growth (usually 2-6x10^5^ parasites/mL) [14].

### Desiccation of the liquid in the VDRL wells

In order to estimate desiccation of the LIT medium at room temperature (20–25°C), each one of 12 VDRL test wells in 4 different plates, was filled with 100μL of LIT medium, thus each plate had a total volume of 1200μL. At four different times (0, 55, 120 and 270 minutes) one of the plates was used to measure the total volume and estimate the desiccation process. The results were compared with two separate experiments in which the activity of the Biospeckle pattern of a well with parasites in LIT medium and a well with LIT medium without parasites was taken at different times, but within a similar period of time as the experiment for the desiccation of the liquid. Assay 1 was performed with and without 2.4x10^4^ parasites per well and the activity of the Biospeckle pattern was taken at 8 different times (1, 18, 35, 52, 70, 90, 107 and 120 min), recording 16 videos, two at each time point. Assay 2 was performed with 4x10^4^ parasites per well and the activity of the Biospeckle pattern was taken at 4 different times (1, 61, 128 and 204 min). The data of this assay correspond with the benznidazol experiment discussed below.

### Effect of parasite quantity

The ability of the method to discriminate different parasite quantities was tested in two ways. In the first experiment, parasites were adjusted to 4x10^4^ parasites/mL, and each well of the VDRL plate was filled with 100μL of LIT medium containing different amounts of the parasite (from 0 to 4x10^3^ parasites/100μL), varying the density of the parasites within the 100μL of each well. In the second experiment, parasites were adjusted to 4x10^5^ parasites/mL, and each well of the VDRL plate was filled with different amounts of LIT medium, changing the volume of liquid within each well (from 13.3 to 133μL), thus varying the amount of parasites (from 0.532x10^4^ to 5.32x10^4^ total parasites per well), while keeping the density constant (4x10^5^ parasites/mL). In each case a video was recorded (10 total videos). This assay was designed as is shown in Table 1. In both experiments, the activity of the Biospeckle pattern was taken using the three approaches for image processing described below.

**Table 1:** Conditions of the Constant and Variable Density Experiments.

Number of well	Constant density				Variable density		
	Height of the liquid column (mm)	Volume of liquid (μL)	Number of parasites per well (x10^4^)	% of the Object’s Plane	Height of the liquid column (mm)	Volume of liquid (μL)	Number of parasites per well (x10^3^)
1	0.10	13.3	0.532	12.48	0.75	100	0
2	0.25	33	1.32	5.03	0.75	100	1
3	0.56	66	2.64	2.52	0.75	100	2
4	0.75	100	4.00	1.66	0.75	100	3
5	1.00	133	5.32	1.25	0.75	100	4

Since the object’s plane has a constant volume of 1.66μL due to the parasite’s dimensions (length 20μm and width 5μm, mean 12.5μm), the percentage that it represents in the constant density experiment increases as the total volume of liquid in the well decreases. With the results of the constant density experiment, the activity of the Biospeckle pattern was evaluated in terms of the percentage of the object’s plane.

### Statistical analysis of the activity of the Biospeckle pattern as a function of the parasite quantity and of the concentration of benznidazole

In order to assess the statistical robustness of the quantitative assay, the Biospeckle pattern of repetitions of three parasite concentrations was evaluated and compared to the Biospeckle pattern of LIT medium without parasites. For this purpose, 6 different wells with LIT medium and 2x10^4^ parasites per well, 6 different wells with LIT medium and 3x10^4^ parasites per well, 9 different wells with LIT medium and 4x10^4^ parasites per well and 11 different wells with LIT medium without parasites from different experiments, were evaluated. In each case a video was recorded (32 total videos) and the activity of the Biospeckle pattern was calculated in each case. The statistical analysis was performed with ANOVA, Tukey *post hoc* and Descriptive Statistics using R and R Commander. For the ANOVA model the null hypothesis states that the mean of the four groups is equal. Therefore, the alternative hypothesis states that at least the mean of one of the four groups is statistically different. The Tukey *post hoc* test is performed if the null hypothesis of the ANOVA Model is rejected, in order to find the combinations by pairs that have statistically different means. R and R Commander were used to perform these tests. A similar analysis was performed with the data of the activity of the Biospeckle pattern as a function of the concentration of benznidazole, with and without parasites: descriptive statistics, ANOVA, Tukey *post hoc*, Bartlett’s test of homogeneity of variances and Levene's test for homogeneity of Variances.

### Effect of different quantities of benznidazole on the activity of the Biospeckle pattern of *Trypanosoma cruzi*

Benznidazole was used as a trypanocidal reference drug. It was provided as a generic drug by the Health Public System in Venezuela. Each well of the VDRL plate was filled with 100μL of LIT medium in the presence or absence of 4x10^4^ epimatigotes per well, containing 7 different concentrations of benznidazole: 0, 1.95x10^-2^, 7.81 x10^-2^, 31.25 x10^-2^, 1.25, 5 and 20 μg/mL (ranging from 0.075 to 76.8μM) and at different times (1, 61, 128 and 204 minutes) a video was recorded (28 videos with parasites and 24 videos without parasites) and in each case the activity of the Biospeckle pattern was calculated. In the plots with a logarithmic scale the 0 concentration values are omitted. In some cases the concentrations are presented with either two or more decimals.

### Experimental setup

**Fig 1:**
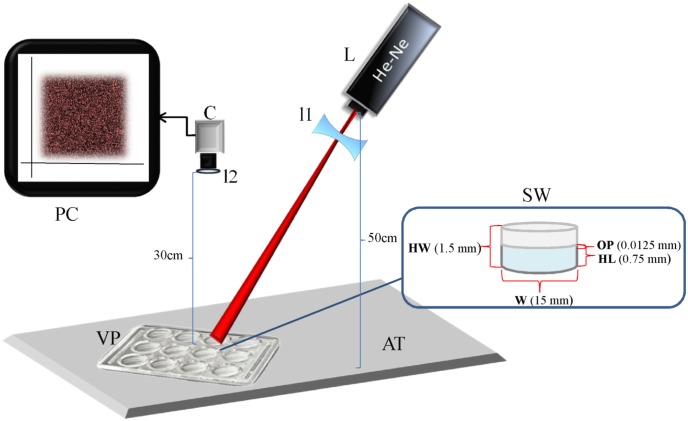
Schematic diagram of the speckle experimental setup. Experimental setup, with data of the setup and the VDRL well, L laser; C CCD camera; PC computer; l1 lens of the laser; l2 lens of the camera; AT anti-vibration table; VP VDRL plate; SW sample well; OP object’s plane; W width; HL height of the liquid; HW height of the well. The diagram is not drawn to scale.

Fig 1 shows the scheme of the experimental setup. The laser Biospeckle imaging system consists of a 1mW He-Ne laser (unpolarized) operating at 632.8nm which is coupled to a convex divergent lens to form a spot of approximately 10mm onto individual wells in the VDRL plate. The laser is located at a distance of 50cm and the beam has an angle of incidence of 72°. Since the wells have an internal diameter of 12.7+/- 0.1mm and the laser beam makes a spot that has a maximum diameter of 10mm, the beam illuminates the center of the well thus avoiding edge effects. The VDRL plate is put on top of an opaque black cardboard fixed on an anti-vibration table. A CCD camera (Thorlabs USB.2, 30 fps, 6.45-μm Square Pixels) is located at 30 cm from the sample and is connected to a PC which records 0.5–1 min videos. The resolution of the camera is 1280 × 1024 Pixels, and its optical system consists in fixed focal lengths of 3.5–75mm with maximum aperture of up to f/0.95, as well as an 18–108 mm f/2.5 zoom lens. The high magnification zoom lenses are made up of a modular system that features magnification from 0.07 to 28. The speckle video data is sent to the computer for video and image processing. With this set up, the illuminated region completely occupies the image in a homogeneous manner so that the whole image is subject to analysis and processing. Fig 1 shows the dimensions of the VDRL well.

### Acquisition and handling of image data

In order to assign numerical values to measure the activity of the dynamic Biospeckle patterns, three approaches were taken, which are based on modifications of the Temporal Difference Method [8]. In all cases, whole successive images of a video were processed. This could be achieved because the setup was designed so that the active region of the image occupies the whole frame in a nearly homogeneous manner so as to be able to consider and compare whole images.

In the first approach, Eq (1) was developed as an algorithm of averages that analyzed all the frames in one video sequence. The frames were downloaded using a frame-by-frame mode in the image processing unit and transformed to a gray scale. Eq (2) was used for the purpose of comparison with Eq (3). In both cases, ***A*** and ***B*** are measures of mean intensity (I), ***A*** being expressed as an averaged mean intensity and ***B*** being expressed as the sum of the mean intensities:

$$A=\sum_{n=1}^{N}\langle |I_{n}-I_{n+1}|\rangle /N-1$$ (1)

$$B=\sum_{n=1}^{N}\langle |I_{n}-I_{n+1}|\rangle$$ (2)

where |.| is the absolute value, <.> is the mean value, I is intensity in gray scale and N represents 1500 total frames analyzed. In the case of Eq (1), the mean intensity of the absolute value of a subtraction pixel by pixel of whole successive images, is averaged to obtain *A*, when a video has 1500 images, there will be 1499 mean differences (N-1). With this algorithm, successive images are subtracted, transformed to the absolute value of the difference, the mean for the absolute values of the intensities of the differences is obtained and finally the means are averaged. It should be noted that with this algorithm, the final number corresponds to the average of means. In the case of Eq (2) the same algorithm is performed adding the mean intensities of the differences, without the final average. In the case of Eq (1), this was achieved with a free software program, ***Image Delta Processor*** (named in this work as ImageDP), written for this purpose in Java, which is provided as Supporting Information (S1 Text Documentation ImageDP, S2 Text Source ImageDP, S3 Text Settings ImageDP, S4 Text Project ImageDP, S5 Text Classpath ImageDP). Eq (2), since it was only used for testing the linearity of the method, it was worked out using the program ImageJ.

In the second approach, Eq (3), a modified version of Eq (2) was used applying the program ImageJ (a public domain Java image processing program developed at NIH, USA). In the third approach, a script using **R Commander/RSAGA/SAGA GIS** (named in this work as R/SAGA) was designed with the same Eq (3). R is a free software environment, R commander is its graphical user interface and SAGA-GIS (System for Automated Geoscientific Analysis-Geographic Information System) is a Free Open Source Software. This software is provided as Supporting Information (S6 Text Script R/SAGA, S7 Text Manual R/SAGA). ***C*** is a measure of the mean intensity (I), being expressed as the sum of the intensities, as explained below:

$$C=\sum_{n=1}^{N-1}\left|I_{n}-I_{n+1}\right|$$ (3)

With ImageJ, a set of frames was downloaded, typically 11 whole frames were taken. The addition of the difference between the consecutive frames leads to the construction of a new image from which the mean intensity (I) is taken. It should be noted that in this case, 11 successive whole frames are subtracted creating 10 new images containing the absolute values of the intensities of the differences, then the 10 new images are added to obtain a resulting image from which the mean intensity is taken. The same algorithm is carried out with R/SAGA for which a set of 11 frames is imported with SAGA and processed in a Linux environment. Then, in a second step with R Commander 10 new images are created by taking the absolute value of the difference between consecutive frames. It should be noted that only “Channel 1” is taken to calculate the absolute value of the difference, so as to exclude two of the color channels or bands and therefore, to make the calculation in gray scale. Finally, the 10 difference images are imported and added in SAGA, generating a final image from which the mean intensity is taken. In this work the activity of the Biospeckle pattern refers to the values of *A*, *B* or *C*, depending on the equation that is selected for calculation. Also, since very different programs and algorithms are used, the absolute values may be different but the relations are comparable. The frames for calculation were sometimes selected from a segment of the video avoiding vibrations from stirring or external interferences but they usually corresponded to the region that goes from more than 3 seconds (to allow stabilization) to less than 25 seconds.

In order to test the linearity of each equation, in one case up to 100 frames of the same video were processed by the algorithm of the three equations with ImageJ and by the algorithm of Eq (3) with R/SAGA. In all these cases, the same set of frames was processed from a video with a high activity of the Biospeckle pattern from the experiment with parasites and benznidazole (0.3125μg/mL or 1.2μM) taken at 1min. Also, as a comparison, a set of frames was taken from a video with a low activity of the Biospeckle pattern from the experiment with benznidazole (0.3125μg/mL or 1.2μM) without parasites taken at 1min, and processed with Eq (3) with R/SAGA and with ImageJ. In order to illustrate the difference in the activity of the Biospeckle pattern, 2, 10 or 30 frames were included in the calculation of 1, 9 or 29 image differences with ImageJ and Eq (3) for the videos of high and low activity of the Biospeckle pattern, respectively and six surface plots were constructed.

## Results

### Effect of parasite quantity

**Fig 2:**
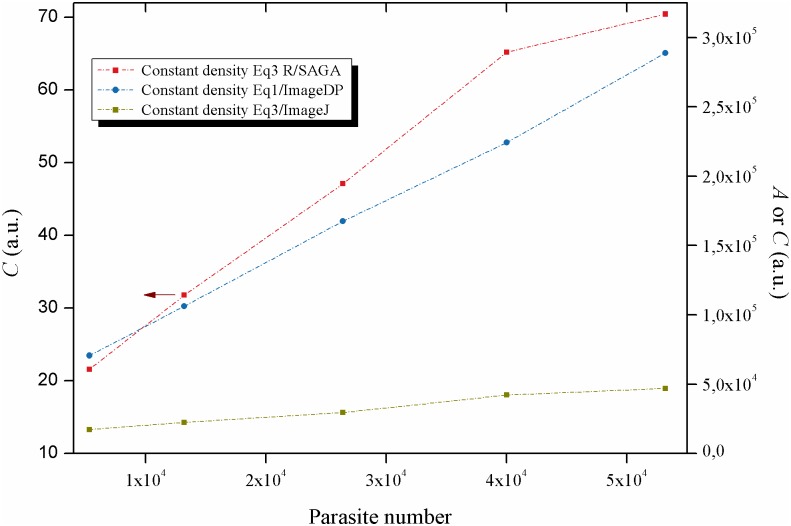
Effect of parasite quantity on the activity of the Biospeckle pattern: Constant density. Constant density experiment, analyzed for image processing with R/SAGA and Eq (3), ImageJ and Eq (3) and ImageDP and Eq (1).

The effect of parasite quantity on the activity Biospeckle pattern was evaluated by conducting two experiments (constant density and variable density) and analyzing in each case, the same video with the three approaches for handling of image data. In the experiment of constant density 5 different test wells had 4x10^4^ parasites/mL but the volume in each well varied from 13.3 to 133μL and the parasite number varied from 0.532 to 5.32x10^4^ parasites per well. As is shown in Fig 2, the three approaches are able to detect an increase in the activity of the Biospeckle pattern as a function of parasite quantity. Moreover, the plots are nearly linear using the three approaches. In the three cases, an increase in the number of parasites obtained by increasing the volume and thus the height of the liquid column in the assay well (constant density), shows linearity. This indicates that if the density is kept constant, the Biospeckle pattern is proportional to the quantity of parasites in the test wells within the evaluated range. In other words the Biospeckle pattern increases as a function of parasite quantity, despite the fact that the density of the parasites in the test wells and in the object’s plane is constant. Therefore, the system is detecting the parasites in the whole well.

**Fig 3:**
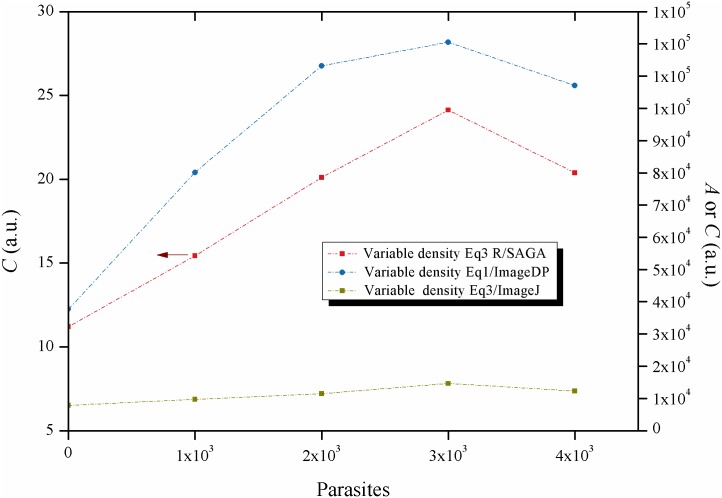
Effect of parasite quantity on the activity of the Biospeckle pattern: Variable density. Variable density experiment, analyzed for image processing with R/SAGA and Eq (3), ImageJ and Eq (3) and ImageDP and Eq (1).

In the experiment of variable density 5 different test wells had 100μL of LIT medium containing from 0 to 4 x10^3^ parasites per well. With two of the approaches (R/SAGA and ImageJ with Eq (3) (Fig 3), a linear relationship is obtained up to 3x10^3^ parasites in the assay well. Moreover, comparing the constant and variable density experiments, a linear behavior is observed in both cases. In contrast with this finding, with the approach ImageDP, an increase in the quantity of parasites by increasing the density and thus the amount of parasites in the object’s plane shows a non-linear behavior. This lack of linearity of ImageDP was used as one of the criteria to exclude this approach as the selected image processing method for the following experiments.

**Fig 4:**
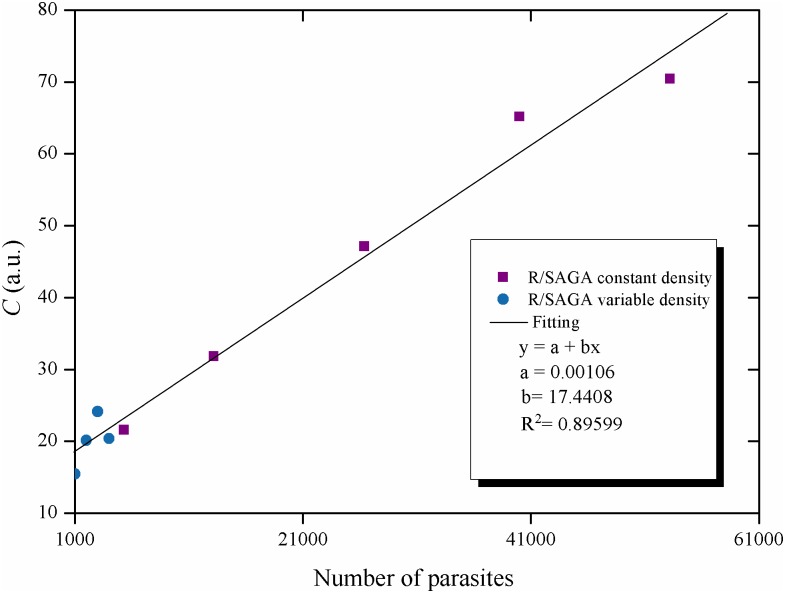
Effect of parasite quantity on the activity of the Biospeckle pattern: Constant and variable density values. Activity of the Biospeckle pattern as a function of the number of parasites, the data were obtained from the constant and variable experiments processed with R/SAGA and Eq (3).

With the other two methods, either ImageJ or R/SAGA with Eq (3), the activity of the Biospeckle pattern of both experiments is a function of the quantity of parasites within the well. Since the experiment for variable density is performed with 0 to 4 x10^3^ parasites in 100μL per well and the experiment for constant density is performed with 0.532 to 5.32x10^4^ parasites in a variable volume per well, and if the method is able to detect the total number of parasites in the well irrespective of the volume, then it should be possible to find a good relation between the activity of the Biospeckle pattern as a function of the number of parasites. Fig 4 shows that the number of parasites of both experiments can fit a linear regression.

### Evaluation and selection of the image processing method for *T*. *cruzi* in the wells of the VDRL plate

**Fig 5:**
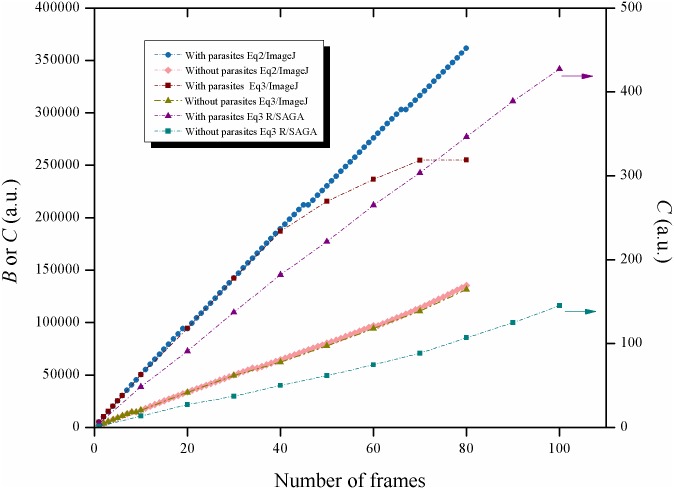
Comparison of the image processing methods: Eqs (2) and (3). Consecutive frames were taken from a video of a well with 4x10^4^ parasites in LIT medium and benznidazole 0.3125μg/mL and frames 100 to 180 were processed with ImageJ using either Eqs (2) or (3); consecutive frames were taken from a video of a well with LIT medium and benznidazole 0.3125μg/mL without parasites and frames 100 to 180 were processed with ImageJ and Eq (3); consecutive frames were taken from a video of a well with 4x10^4^ parasites in LIT medium and benznidazole 0.3125μg/mL and frames 100 to 200 were processed with R/SAGA and Eq (3); consecutive frames were taken from a video of a well with LIT medium and benznidazole 0.3125μg/mL and without parasites and frames 100 to 200 were processed with R/SAGA and Eq (3).

**Fig 6:**
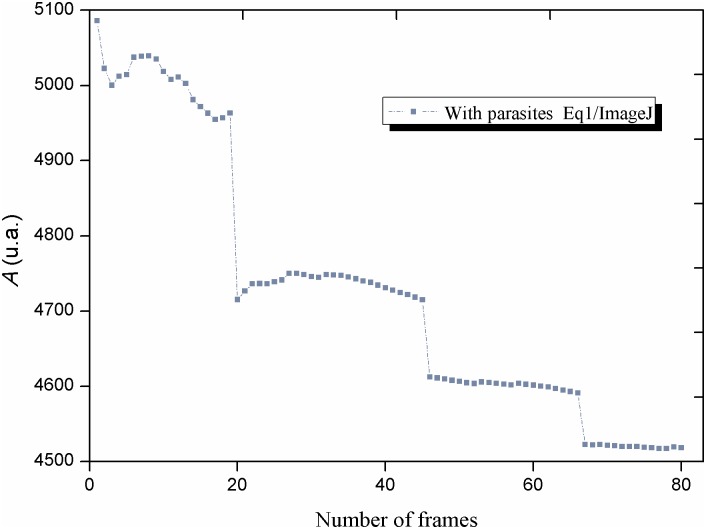
Comparison of the image processing methods: Eq (1). Consecutive frames were taken from a video of a well with 4x10^4^ parasites in LIT medium and benznidazole 0.3125μg/mL and frames 100 to 180 were processed with ImageJ and Eq (1).

**Fig 7:**
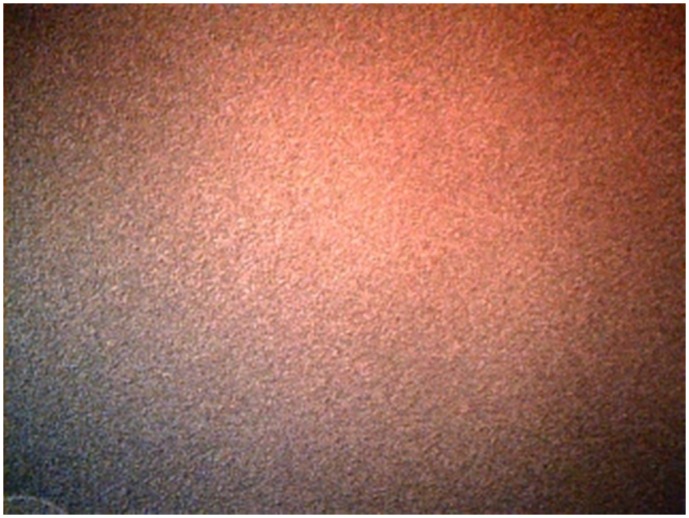
High activity of the Biospeckle pattern. Photogram taken from a video of a well with 4x10^4^ parasites in LIT medium and benznidazole 0.3125μg/mL at 1min.

**Fig 8:**
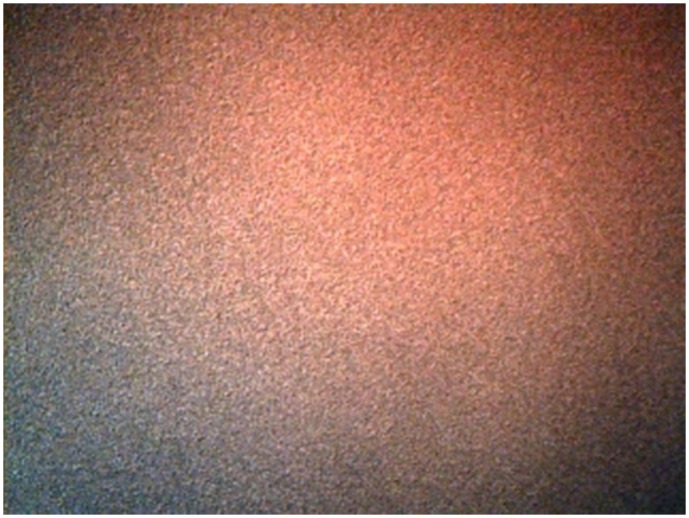
Low activity of the Biospeckle pattern. Photogram taken from a video of a well with LIT medium and benznidazole 0.3125μg/mL without parasites at 1min.

In order to further examine the proposed equations, a comparison of algorithms and software was performed, as is shown in Figs 5 and 6. Since ImageDP uses all the photograms in a video (750–1500 frames), in order to compare the result of using the equations on a selected set of photograms (up to 100 frames), only ImageJ or R/SAGA and not ImageDP, were used to analyze the same segment of a video. Figs 7 and 8 show two frames taken from a video with high Biospeckle activity and low Biospeckle activity, respectively.

**Fig 9:**
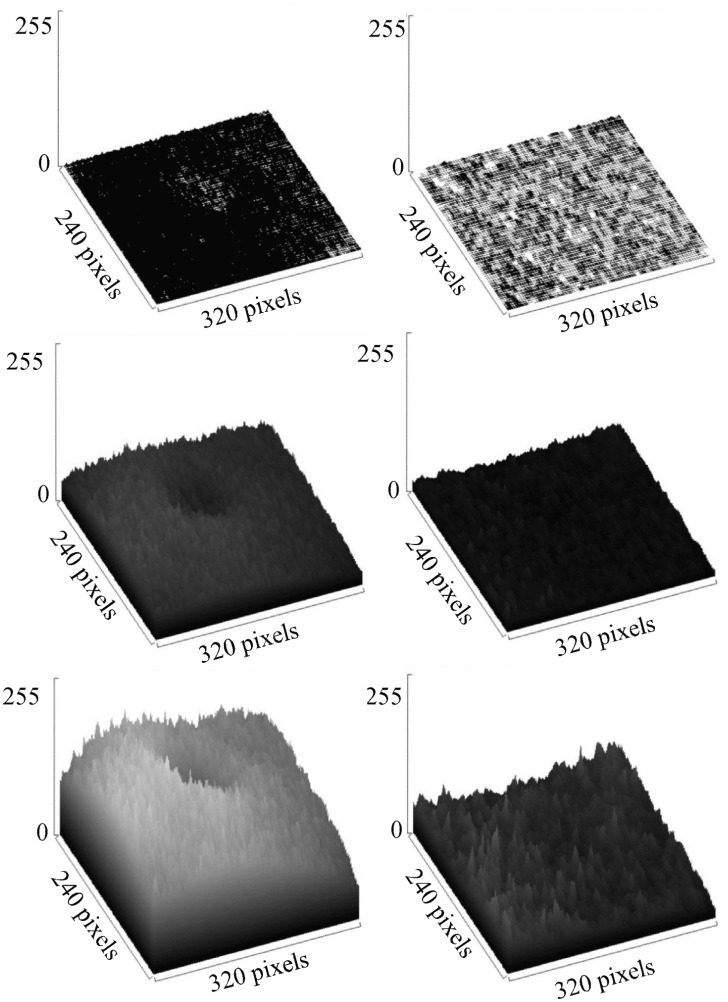
Surface plots comparing a video with high Biospeckle activity and a video with low Biospeckle activity. (a),(b) and (c) Surface plots of the images that were obtained from a video with parasites and benznidazole 0.3125μg/mL at 1min, calculated with ImageJ and Eq (3) in Fig 5, with 2, 10 and 30 frames (1, 9 and 29 image differences) included in the calculation, respectively. (d),(e) and (f) Surface plots of the images that were obtained from a video without parasites and benznidazole 0.3125μg/mL at 1min, calculated with ImageJ and Eq (3) in Fig 5, with 2, 10 and 30 frames (1, 9 and 29 image differences), included in the calculation, respectively.

Using the program ImageJ, Eqs (2) and (3) show similar curves when the calculation includes up to 40 frames (Fig 5) but if more frames are included there is a saturation effect when using Eq (3). Since both equations were worked out with the same program (ImageJ), the difference between the curves must be due to the algorithms. In the case of Eq (2), *B* corresponds to the sum of numbers, each one representing the mean obtained after subtracting consecutive frames. In the case of Eq (3), *C* corresponds to the mean intensity of an image that was constructed by adding the images that resulted by taking the difference between consecutive frames. Therefore, in the former, there is the addition of numbers while in the latter there is the addition of image rasters, showing that in this case, there is a saturation with non-linear behavior as more frames are included in the calculation, possibly due to the fact that this is an image processing program with a radiometric resolution with a limit of intensity at a value of 256. Fig 5, also shows that if the video has a lower activity of the Biospeckle pattern as would be expected in the case of LIT medium without parasites, there is no saturation with the same number of frames, suggesting that the number of frames required to reach this saturation effect depends on the activity of the Biospeckle pattern of the selected video. This effect is further analyzed in Fig 9 which shows the surface plots of the images constructed for the curves obtained with ImageJ and Eq (3) of Fig 5, with 2, 10 and 30 frames (1, 9 and 29 image differences) included in the calculation, avoiding the saturation that occurs after 40 frames. A marked difference is observed when comparing the surface plots on the right (Fig 9.d, 9.e and 9.f) which were obtained with LIT medium without parasites, with the surface plots on the left (Fig 9.a, 9.b and 9.c) which were obtained with LIT medium with parasites, with the same concentration of benznidazole, in both cases. As is shown in the left panel of Fig 9, the presence of parasites increases the activity of the Biospeckle pattern, which explains the saturation that is obtained in Fig 5. Conversely, as is shown in the right panel of Fig 9, the absence of parasites shows low activity of the Biospeckle pattern, which explains that there is no saturation (Fig 5), at least in the tested interval.

As shown in Fig 5, with Eq (2) a linear behavior is obtained up to at least 80 frames, however, if this algorithm is worked out as Eq (1) as would be the case for the program ImageDP, performing the average, the value tends to decrease as more frames are included in the calculation for image processing (Fig 6). Since the program ImageDP was designed for Eq (1) and for processing all the frames in a video, we assume that the final value (*A*) will be affected by this decrease and will be a fraction of the value obtained at the beginning of the video. This observation could explain the shape of the curve of variable density of parasites (Fig 3). Therefore, up to this point the results indicate that we are obtaining only approximate values because the algorithm of Eq (1) with ImageDP calculates decreased values and the algorithm of Eq (3) with ImageJ has the tendency to reach saturation. However, as is shown in Fig 5, Eq (3) works well with R/SAGA, in both cases with and without parasites, showing a linear relation up to 100 frames. Since usually no more than 20 frames are selected for the calculation, it can be expected to provide an adequate representation of the Biospeckle pattern and this is in agreement with the curves for constant and variable density of parasites of Figs 2 and 3. Therefore, in the following experiments of this work, all the calculations were carried out using Eq (3) with R/SAGA.

### Effect of the object’s plane

**Fig 10:**
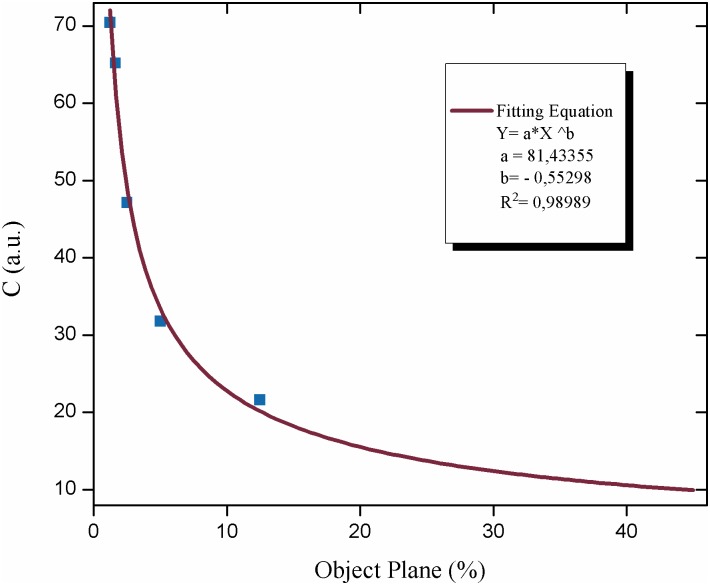
Effect of the object’s plane on the activity of the Biospeckle pattern. The change in the activity of the Biospeckle pattern calculated by R/SAGA and Eq (3) is shown as a function of the percent of the object’s plane. The data were taken from the constant density curve of Table 1 and Fig 2.

In order to test the relationship between the object’s plane and the activity of the Biospeckle pattern, we used the data of Table 1 and the constant density curve of Fig 2. Fig 10 shows the activity of the Biospeckle pattern (with R/SAGA Eq 3) as a function of the increase of the calculated percentage of the volume of the disk of the object’s plane with respect to the total volume of the liquid in the well, in the experiment in which the total volume decreases. It shows that the intensity decreases as the percentage of the disk increases, indicating that as it was stated above, since the density is constant and the percentage of the object’s plane increases, the system is detecting the total amount of parasites in the well.

### Statistical analysis of the activity of the Biospeckle patterns as a function of the parasite quantity

Applying an ANOVA Model on the activity of the Biospeckle pattern on 32 values of four groups of parasites per well (0, 2x10^4^, 3x10^4^ and 4x10^4^), the null hypothesis stating that the mean of the four groups is equal, is rejected because the p-value (2x10^-16^) is less than the significance level of 0.05. As is shown in Supporting Information (S1 Data Statistical Analysis), the assumptions for normal distribution, homoscedasticity of the residuals and absence of autocorrelation, are satisfied.

Thus a Tukey *post hoc* test was carried out in order to explore the differences among the four groups of data, compared in pairs. As Table 2 shows, all the p-values for the six combinations are less than the significance level of 0.05 which indicates that the mean value between pairs, is statistically different.

**Table 2:** Tukey *post hoc* test (see complete data in S1 Data).

Combination (Parasites per well)	p-value
2x10^4^–0	0
3x104–0	0
4x104–0	0
2x10^4^–3x10^4^	0.046
2x10^4^–4x10^4^	0
3x10^4^–4x10^4^	0

p-value: probability of Error Type I.

Finally, Descriptive Statistics tests were performed on each group of data, obtaining the results shown in Table 3, where parameters such as Mean and Standard Deviation of each group can be obtained, as well as the indicators of the type of distribution of the data.

**Table 3:** Descriptive statistics tests (see complete data in S1 Data).

	M	SD
P0	21.97	3.77
P20000	38.59	2.09
P30000	44.74	1.88
P40000	58.27	5.41

M: Mean, SD: Standard Deviation.

### Effect of desiccation

**Fig 11:**
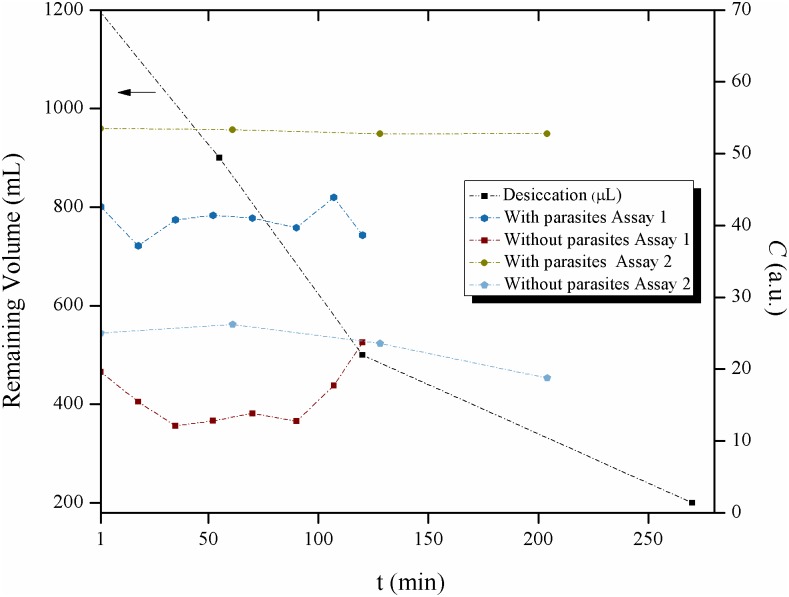
Effect of desiccation of the liquid within the wells of the VDRL plate. Remaining volume after desiccation (μL) or activity of the Biospeckle pattern (*C*), as a function of time (min).

The desiccation of the liquid in the air exposed VDRL wells and its implications on the activity of the Biospeckle pattern was evaluated in several ways. Fig 11 shows the desiccation in terms of remaining volume, after 0, 55, 120 and 270 minutes have elapsed. In the same Figure two assays (Assay 1 and Assay 2) show the activity of the Biospeckle pattern in separate wells with and without parasites and processed under R/SAGA and Eq (3). The results indicate that although there is a marked desiccation effect, the Biospeckle pattern remains stable up to around 200 minutes, indicating that as was seen in Figs 2, 3 and 4 (Effect of Parasite Quantity), the Biospeckle pattern under the conditions of this work, reveals the total number of parasites within the well of the VDRL plate. However, it should be noted that Assay 1 shows a more variable profile than Assay 2, suggesting that the lower number of parasites and the repeated irradiation of the same sample well with the laser beam may have an effect on the activity of the Biospeckle pattern, in both cases, with and without parasites.

### Selection of the conditions for the benznidazole assay

These assays allowed the selection of the conditions of the method using VDRL plates. We selected the following conditions: 100μL in the test well, parasites in exponential phase, 4x10^4^ parasites per well, a maximum time of evaluation of the activity of the Biospeckle pattern of 204 minutes. The recommended method for image processing is R/SAGA with Eq (3).

### Effect of different quantities of benznidazole on the activity of the Biospeckle pattern of the parasites

**Fig 12:**
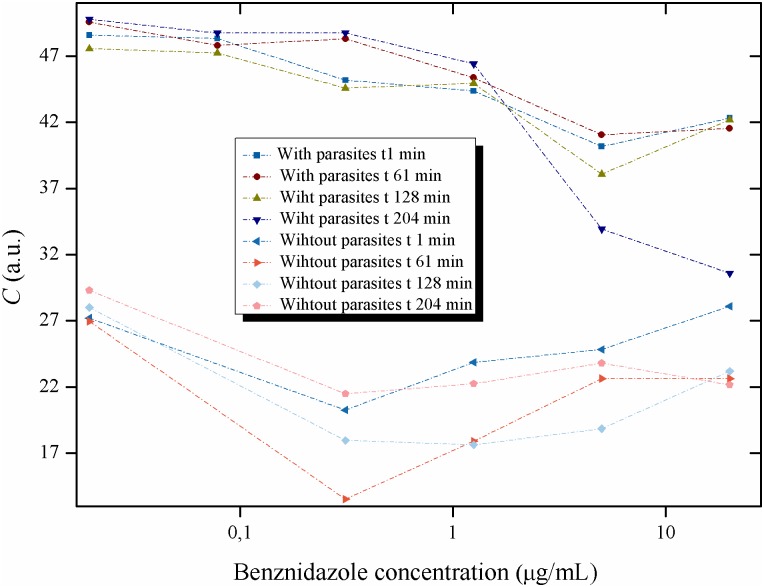
Activity of the Biospeckle pattern of *Trypanosoma cruzi* in LIT medium as a function of the concentration of benznidazole: with and without parasites. Activity of the Biospeckle pattern of 4x10^4^ epimastigotes of *T*. *cruzi* as a function of several concentrations of benznidazole (0.0195; 0.0781; 0.3125; 1.25; 5 and 20μg/mL) and at different times (1, 61, 128 and 204min), compared with LIT medium without parasites with the same concentrations of benznidazole (except 0.0781 μg/mL) and at the same times.

**Fig 13:**
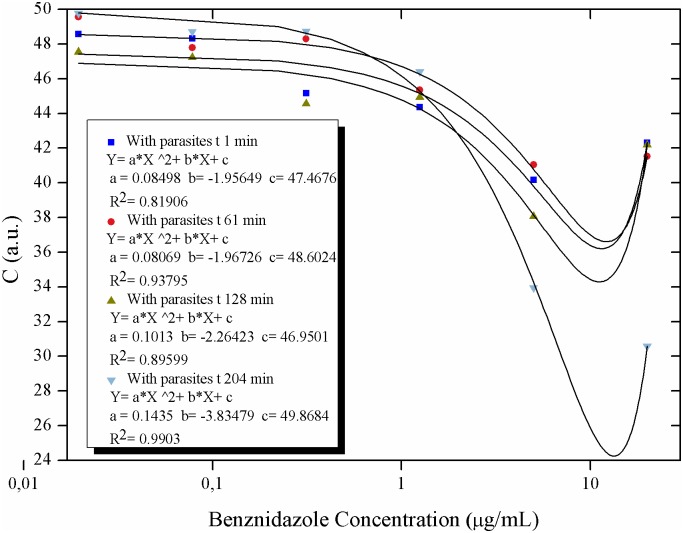
Activity of the Biospeckle pattern of *Trypanosoma cruzi* in LIT medium as a function of the concentration of benznidazole: polynomial regression curves. Polynomial regression curves for the activity of the Biospeckle pattern of 4x10^4^ epimastigotes of *T*. *cruzi* as a function of several concentrations of benznidazole (0.0195; 0.0781; 0.3125; 1.25; 5 and 20μg/mL) and at different times (1, 61, 128 and 204min).

**Fig 14:**
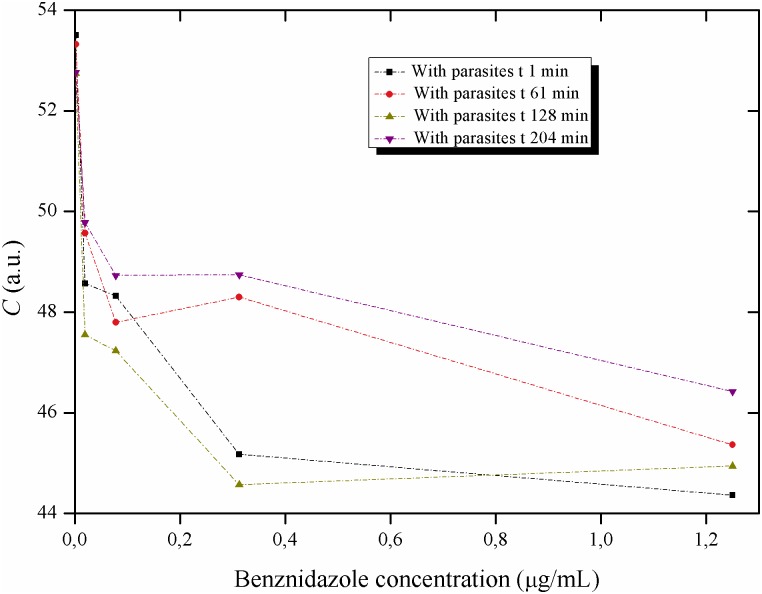
Activity of the Biospeckle pattern of *Trypanosoma cruzi* in LIT medium as a function of the concentration of benznidazole: low concentrations of the drug. Activity of the Biospeckle pattern of 4x10^4^ epimastigotes of *T*. *cruzi* as a function of several concentrations of benznidazole (0; 0.0195; 0.0781; 0.3125 and 1.25μg/mL) and at different times (1, 61, 128 and 204min).

**Fig 15:**
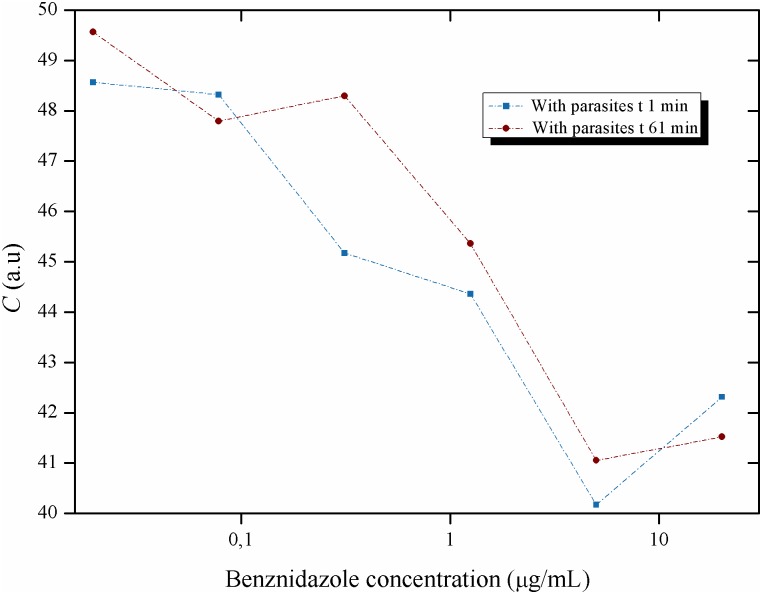
Activity of the Biospeckle pattern of *Trypanosoma cruzi* in LIT medium as a function of the concentration of benznidazole: early activity of the drug. Activity of the Biospeckle pattern of 4x10^4^ epimastigotes of *T*. *cruzi* as a function of several concentrations of benznidazole (0.0195; 0.0781; 0.3125; 1.25; 5 and 20μg/mL) and at times 1and 61min.

**Fig 16:**
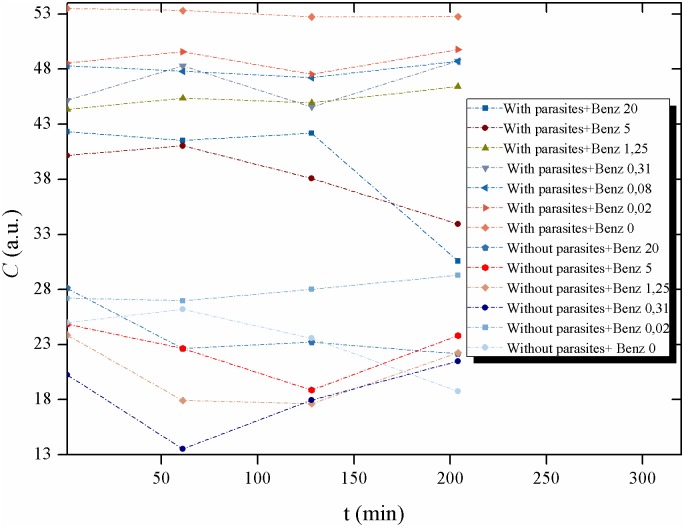
Activity of the Biospeckle pattern of *Trypanosoma cruzi* in LIT medium as a function of time: with and without parasites. Activity of the Biospeckle pattern of 4x10^4^ epimastigotes of *T*. *cruzi* as a function of different times (1, 61, 128 and 204min) at several concentrations of benznidazole (0; 0.0195; 0.0781; 0.3125; 1.25; 5 and 20μg/mL) and compared with LIT medium without parasites at the same times with the same concentrations of benznidazole (except 0.0781 μg/mL).

With these conditions, the effect of varying amounts of benznidazole on the activity of the parasites was tested and compared to the pattern obtained from the LIT medium with benznidazole and without parasites. Videos of each well were taken at different times. Figs 12, 13, 14, 15 and 16 show the curves obtained for the activity of the Biospeckle pattern of 4x10^4^ parasites per well, at times of 1, 61, 128 and 204 minutes with six concentrations of benznidazol ranging from 0.0195 to 20μg/mL, and as a comparison, the curves obtained with benznidazole without parasites. Fig 12 shows that there is a distinct difference between the curves with and without parasites, as would be expected from the statistical analysis (Table 3) in which there was a significant difference among the means for samples with these two conditions. In this case the assays in the presence of growing benznidazole concentrations with and without parasites, show activities of the Biospeckle pattern that are statistically different with means of 46.02±5.27 and 22.76±3.95, respectively (see S1 Data).

The effect of benznidazole on *T*. *cruzi* was analyzed from two perspectives, concentration and time of action of the drug. The activity of the Biospeckle pattern as a function of benznidazole concentration shows curves that fit similar Polynomial regressions at all the tested times (Fig 13) with a decrease of the activity as a function of concentration and a clustering of the values (mean of the clustered low concentration values of 48.39±2.91, see S1 Data for details) of the four time curves up to a concentration of benznidazole of 1.25μg/mL. Fig 14 which includes the activity of the Biospeckle pattern in the absence of benznidazole, shows that at the low concentrations the four time curves have a similar profile. However at greater concentrations of benznidazole (Fig 13), there is a dispersion of the values (mean of the dispersed high concentration values of 40.12±5.33, see S1 Data for details) of the activity of the Biospeckle pattern. In the absence of parasites the mean values are 22.50±4.53 and 23.28±2.61 for the lower and higher concentrations of benznidazole, respectively. As shown in S1 Data, in the presence of parasites the mean value of the activity of the Biospeckle pattern at the high concentrations of benznidazole is statistically different from the mean value of the activity of the Biospeckle pattern at the low concentrations of benznidazole. Conversely, without parasites the means for both groups (low and high concentrations of benznidazole), are statistically equal. All this indicates that the growing concentrations of benznidazole cause an activity window that can be detected by the statistically lower mean value only in the presence of parasites. Moreover, the variability of the four groups is statistically equal as is shown by Bartlett’s test and Levene's test for homogeneity of variances which show p-values that are higher than 0.05, indicating that the variances are statistically equal.

On the other hand the behavior of the activity of the Biospeckle pattern at the shorter times was explored plotting only the curves for 1min and 61min for all the concentrations of benznidazole. As shown in Fig 15, there is an instantaneous effect at 1min that remains with similar activity values at least for one hour for all the benznidazole concentrations. The profile of these two early curves is different from the two late curves (128min and 204min) only at the higher concentrations (5 and 20μg/mL), Fig 13.

Analyzing the time of action of the drug, the same data were used in Fig 16 to evaluate the effect of the elapsed time on the activity of the Biospeckle pattern for each concentration of benznidazol, with and without parasites. In this case the lower values show a higher activity of the Biospeckle pattern than the higher values of benznidazol at all the tested times. Moreover, at the lower concentrations, the activity remains stable up to 204 minutes. At the higher concentrations (5 and 20μg/mL), the activity of the Biospeckle pattern is stable up to 61 minutes and then decreases reaching final values that approach the activity of LIT medium without parasites.

The results of Figs 12, 13, 14, 15 and 16 suggest that benznidazole produces an instantaneous effect at 1min on *T*. *cruzi* that occurs at all the tested concentrations, with a profile that remains almost the same up to at least 61min, as shown by the polynomial tendencies (Fig 13) and the resulting profile of Fig 15.

Furthermore, the activity of the Biospeckle pattern is similar for all the tested times up to a concentration of 1.25μg/mL (4.8μM). At the higher concentrations of 5 and 20μg/mL (19.2 and 76.8 μM, respectively) the elapsed time seems to have a more relevant effect on this activity. This reveals that there are two effects on *T*. *cruzi*, one at the lower concentrations and another at the higher concentrations. The former is less affected by the elapsed time than the latter, which could be explained by the combination of elapsed time and higher concentrations of the drug. It can be assumed that desiccation may interfere by increasing drug concentration at the longer times. However the activity of the Biospeckle pattern at 1.25μg/mL is similar at all the tested times in spite of the fact that desiccation affects each time curve differently. The highest benznidazole concentrations (20μg/mL, 76.8μM) could be interfering with the assays that were performed at 1, 61 and 128min. At 204min it is possible that this interference caused by the high concentration of the drug may be neutralized by a decrease of the activity promoted by the elapsed time.

As these results show, the present method reveals in a relatively short time, that the effect of benznidazole is dependent on the elapsed time and concentration of the drug, as has been demonstrated by other authors in assays that require a much longer time [3].

A closer look at Figs 12, 13, 14, 15 and 16 reveals that all the time curves have the smallest value of the activity of the Biospeckle pattern in a range that goes from 5 to 20μg/mL (19.2 to 76.8μM, respectively). This range of values represents the concentration of benznidazole that gives maximal inhibition under the assayed conditions. However, 1.25μg/mL (4.8μM) could be considered as the inflexion point that suggests the existence of a minimal concentration that affects the activity of the Biospeckle pattern, separating the effect of benznidazole at low concentrations from the effect at higher concentrations. Traditional IC50 determination requires a measure of the viability of the organism as a function of concentration of the tested drug. In this work the Biospeckle pattern is taken as a measure of the activity of the parasites and the relation of this pattern to viability and drug action has to be determined. Rodrigues *et al* [3] report approximately 12μM for the lowest survival index and an IC50 value of 8.8±0.04μM for benznidazole acting on epimastigotes in LIT culture medium in a period of 96 hours. It is important to point out that in the traditional *in vitro* assays with *Trypanosoma cruzi* [4], benznidazole is usually used in a range of 0.156 to 5μg/mL (0.6 to 19.2μM), and the parasites are counted after 96 hours. In the present work, the concentration of benznidazole is used in a range of 0.0195 to 20μg/mL (from 0.075 to 76.8μM) and the activity is detected in a minimum time of 1 minute.

## Discussion

In this work we are describing a quantitative research aiming at parametric calibration. In this case, the number of observations *n* is greater than the number of estimated parameters which is in agreement with Gujarati and Potter [15] for the adjustment of a parametric model.

A quantitative method for the evaluation of the activity of *Trypanosoma cruzi*, using relatively small volumes and short times has been designed. VDRL plates were chosen because they are widely known and used for other purposes and assays and because the liquid contained in the assay well is flat shaped and therefore, has a minimal distortion with the optical setup and the recorded video.

For image processing two levels of complexity have been designed. The use of an image processing program such as ImageJ to work out Eq (3) provides a readily available and easy to use system for the analysis of Biospeckle patterns because this is software released by NIH that is in the public domain. However, due to its tendency to reach saturation with the designed algorithm, this method provides only approximate values depending on the intensity of the Biospeckle pattern. Hence, the upper limit of the linear tendency (lower limit of saturation), could be used to qualify the relative Biospeckle activity recorded in a video. Furthermore, the combination of ImageJ with Eq (3) gives approximate values when the Biospeckle pattern is high, but it could be the appropriate choice if the activity of the Biospeckle pattern is low, such as in the case of a low parasite density.

On the other hand, ImageDP and R/SAGA can be used for calculating the values of the Biospeckle pattern, but in both cases a program had to be designed for this purpose. In the case of Eq (1) and ImageDP, the program is suitable for high parasite densities and significant differences among samples, due to its tendency to decrease the real value of the Biospeckle pattern. In contrast, with Eq (3) and R/SAGA a linear profile is obtained. Due to this and to the fact that there is no distortion up to 100 processed frames, R/SAGA is the approach recommended for the evaluation of the activity of *T*. *cruzi* using VDRL plates with the selected conditions that are reported in this work. However, the three approaches tested in this work are useful for the evaluation of the activity of the Biospeckle pattern of *T*. *cruzi* in the VDRL plate. ImageJ with Eq (3) works well if the Biospeckle pattern is low, ImageDP with Eq (1) is suitable for videos with a high activity and R/SAGA with Eq (3) has shown up to now, the best way to describe slight differences of high and low activity videos suggesting that it is the best to test the effect of a drug on a population of parasites.

It should be noted that with the combination of the optical setup and R/SAGA with Eq (3), the system is able to detect all the parasites that are present in the test well, being able to quantify the differences among at least two orders of magnitude. Furthermore, in the tested range, the activity of the Biospeckle pattern is proportional to the number of parasites present within the well, regardless of the amount of liquid which can vary as a result of assay volume or as a result of desiccation. This observation is relevant when considering the action of drugs on the parasites due to the fact that different drugs will have different modes of action, altering the motility, the shape, the aggregation and even the number of live parasites within the well. Since the drug treated parasites will still be present in the well and may still be motile and active, it may be necessary to use an image processing method that is able to detect subtle changes. In the case of R/SAGA since the presence of parasites is significantly different to the absence of parasites as more frames are included in the calculation (Fig 5), it is possible that using up to 80 frames the small differences that are caused by a drug, may be more effectively detected.

Although statistically the method has shown to be reproducible and acceptable for the quantification of *T*. *cruzi* epimastigotes and the differential effect of high and low concentrations of benznidazole on the activity of the Biospeckle pattern, there is much need of improvement in areas such as technical refinement, automation of the algorithms and interaction of interdisciplinary orientations.

Focusing on the parasite, it is important to consider that two modes of motion have been described for *T*. *cruzi* epimastigotes, a persistent (a quasi rectilinear fashion) and a tumbling mode (more complex and difficult to describe) [16]. The selection of the best assay and image processing conditions must take into consideration the assumption that the activity of *T*. *cruzi* epimastigotes could be described by both types of motion under regular culture conditions. The switching between different modes of motion is associated to chemotaxis or some other types of taxis [16]. Thus, although it has not been experimentally proved, the findings in this work suggest that *T*. *cruzi* epimastigotes experience some kind of taxis in the presence of benznidazole which could explain the effect of the drug on *T*. *cruzi* epimastigotes, in a very short time. This statement is in agreement with the suggestion that deviations from a usual motility pattern could be employed as an indicator of drug activity in a drug test [17].

Benznidazole is a pro-drug that has to be converted to the active drug. In *T*. *cruzi* benznidazole is metabolized by an NADH-dependent, mitochondrial localized type I nitro-reductase rendering the cytotoxic and mutagenic agent glyoxal [18] [19]. However, it has also been shown that *T*. *cruzi* has a TcABCG1 transporter protein, located on the plasma membrane for which a nucleotide binding, a membrane and a transmembrane domain have been described. This protein is involved in a variety of translocation processes including benznidazole [20]. Transport processes are rapid and efficient mechanisms and could respond to intracellular and extracellular signals [21]. This probably constitutes the first line of response for pro-drug uptake and could represent a type of taxis which acts in a very short time. From the activity curves for benznidazole on *T*. *cruzi*, we could presume that we are in the presence of a two-step mechanism, one that is an almost immediate response to the presence of benznidazole and another that occurs after the pro-drug has been internalized. Whether the activity of the Biospeckle pattern is detecting these two steps has to be further investigated. Before this, it would be necessary to establish the relationship between viability and the activity of the Biospeckle pattern, the possible optical interferences of high drug concentrations which should be avoided especially in those cases in which desiccation is also an issue to be taken care of, the requirement of this method to measure activity in a period of time that may not be long enough to allow the expression of the complete mechanism of action in order to be comparable with the viability tests performed at 96 hours and the requirement for a metabolic condition of the parasites that should be standardized since this may have implications on motility and aggregation, thus on the activity of the Biospeckle pattern. Also it would be interesting to perform a parametric analysis on trypomastigote/amastigote stages with the proposed method.

Finally, it is observed that with this method it might be difficult to design an experiment to determine an IC50 due to the fact that the meaning of the activity of the Biospeckle pattern with respect to viability is unknown. However, what is known is that, at concentrations of benznidazole above 1.25μg/mL (4.8μM), there are important changes in the profiles of the responses, suggesting that this can be considered as the minimal concentration that affects the activity of the Biospeckle pattern, under these conditions.

## Conclusions

A method has been designed for the evaluation of the activity of the Biospeckle pattern of *Trypanosoma cruzi*, using relatively small volumes, short times, VDRL plates, an optical setup with a laser and a camera and an image processing method with R/SAGA. It is concluded that the activity of the Biospeckle pattern is proportional to the quantity of parasites present in the assay well and although it is susceptible to desiccation, the measure is stable up to around 200 minutes. With this quantitative method it is possible to statistically differentiate the amount of parasites along two orders of magnitude and although with certain limitations, it is possible to evaluate the effect of benznidazole on *T*. *cruzi*. In spite of the limitations, an effect of benznidazole on the activity of the Biospeckle pattern of *T*. *cruzi* epimastigotes can be detected at concentrations higher than 1.25μg/mL which is statistically different from the effect at lower concentrations. This effect is better detected after 1 hour of drug action. Further studies are necessary to elucidate how exactly the activity of the Biospeckle pattern is related to viability and drug effect.

## Supporting Information

S1 Data Statistical Analysis. (PDF)

S1 Text Documentation ImageDP. User’s Manual for the Program **Image Delta Processor** (ImageDP) designed and written in Java for this work.
(PDF)

S2 Text Source ImageDP. Source code for the Program **Image Delta Processor** (ImageDP).
(PDF)

S3 Text Settings ImageDP. Settings for Program **Image Delta Processor** (ImageDP).
(PDF)

S4 Text Project ImageDP. Project for **Image Delta Processor** (ImageDP).
(PDF)

S5 Text Classpath ImageDP. Classpath for **Image Delta Processor** (ImageDP).
(PDF)

S6 Text Script R/SAGA. Script that uses R Commander/RSAGA/SAGA GIS (R/SAGA), designed and written for this work.
(PDF)

S7 Text Manual R/SAGA. User’s Manual for Script R/SAGA.
(PDF)
