# Supplementary material for: Quantitative Laser Biospeckle Method for the Evaluation of the Activity of Trypanosoma cruzi Using VDRL Plates and Digital Analysis
Source: PLoS Negl Trop Dis. 2016 Dec 5;10(12):e0005169. doi: 10.1371/journal.pntd.0005169 (PMC5137869; doi:10.1371/journal.pntd.0005169)
Supplement: S1 Data — (PDF) [file pntd.0005169.s001.pdf]

S1 Data

Statistical Analysis

QUANTITATIVE LASER BIOSPECKLE METHOD FOR THE EVALUATION OF THE ACTIVITY OF *Trypanosoma cruzi* USING VDRL PLATES AND DIGITAL ANALYSIS

Hilda Cristina Grassi, Lisbette C. García, María Lorena Lobo-Sulbarán, Ana Velásquez, Francisco A. Andrades-Grassi, Humberto Cabrera, Jesús E. Andrades-Grassi, Efrén D.J. Andrades

STATISTICAL ANALYSIS.

PART I: Activity of the Biospeckle Pattern as a function of Parasite Number

DATA

2E04 p/well	3E04 p/well	4E04 p/well	0 p/well
Arith Mean	Arith Mean	Arith Mean	Arith Mean
40.748	44.083	54.742	24.996
37.459	45.207	52.98	21.984
35.559	47.330	57.895	19.598
39.022	42.913	65.189	15.453
40.99	46.304	53.829	20.876
37.75	42.608	51.394	17.903
		63.855	26.200
		59.471	25.588
		65.065	23.574
			26.786
			18.757

ANOVA MODEL

NULL HYPOTHESIS: The Means for 0 parasites/well, 2E04 parasites/well, 3E04 parasites/well and 4E04 parasites/well are equal.

ALTERNATIVE HYPOTHESIS: At least the mean of one of the four groups is statistically different.

	Df	Sum Sq	Mean Sq	F-value	p-value
Parasite quantity	3	6719	2239.6	150.8	<2e-16 ***
Residuals	28	416	14-8		

Since the the p-value ($2x10^{-16}$) is less than the significance level of 0.05, the null hypothesis stating that the mean of the four groups is equal, is rejected.

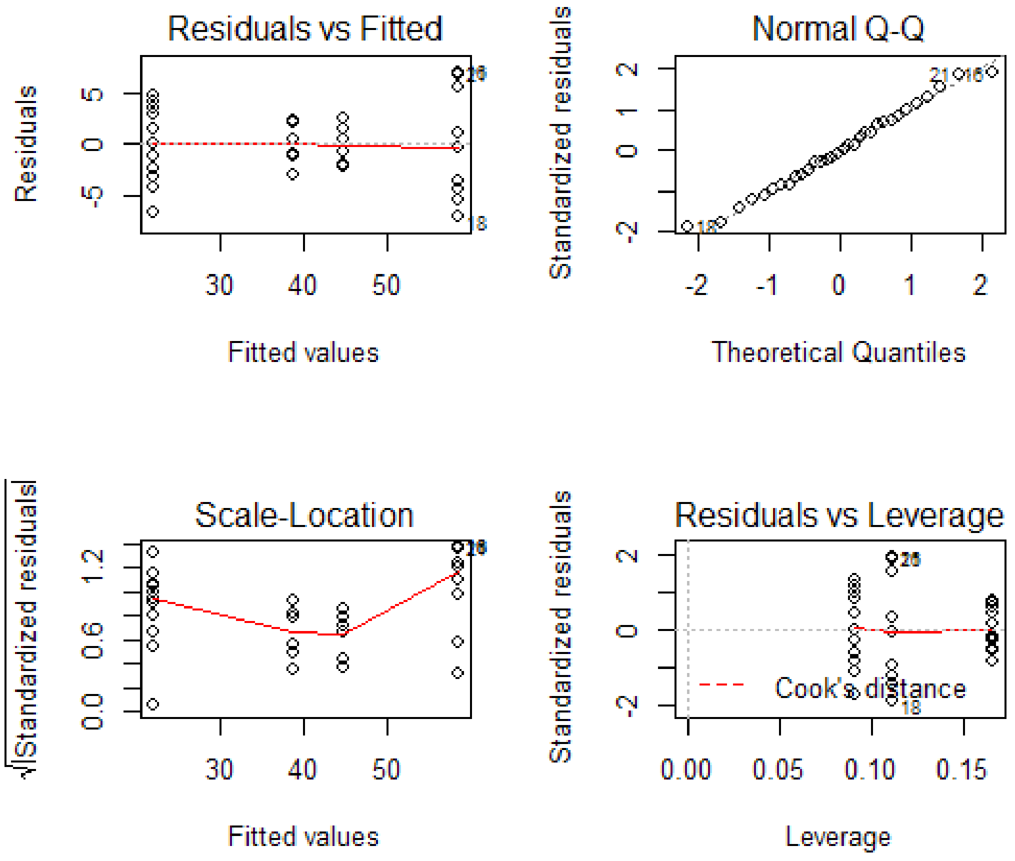

Residuals of the ANOVA Model

Breusch-Pagan	BP	DF	P-VALUE
	1.6801	1	0.1949
Durbin-Watson	DW	DF	P-VALUE
	1.7965	---	0.2541

Residuals indicate the presence of a normal distribution. Therefore it is assumed that the number of observations is sufficient for the number of parameters estimated by the ANOVA Model. Additionally, the BREUSCH & PAGAN Test indicates that there is

homoscedasticity of the residuals and the test for autocorrelation of the residuals indicates that there is no remaining autocorrelation , that is, there is a process of White Noise in the residuals. Due to that, the assumptions of the ANOVA Model are accomplished

TUKEY POST HOC

Since the Null Hypothesis was rejected, a Tukey *post hoc* Test was performed in order to find the combinations by pairs that have statistically different means. All the p-values for the six combinations are less than the significance level of 0.05 which indicates that the mean value between pairs are statistically different.

Combination (Parasites per well)	Diff	Lwr	Upr	p-value
$2x10^4 – 0$	16.61	11.27	21.95	0
$3x10^4 – 0$	22.76	17.42	28.10	0
$4x10^4 – 0$	36.29	31.56	41.02	0
$2x10^4$ - $3x10^4$	6.15	0.078	12.22	0.046
$2x10^4$ - $4x10^4$	19.68	14.13	25.22	0
$3x10^4$ - $4x10^4$	13.52	7.98	19.07	0

Diff: Difference among means, Lwr: Minimum value, Upr: Maximum value, p-value: probability of Error Type I.

DESCRIPTIVE STATISTICS

	M	SD	IQR	VC	Sk	K	0%	25%	50%	75%	100%	DN
P0	21.97	3.77	6.11	0.17	-0.29	-1.13	15.45	19.18	21.98	25.29	26.79	11
P20000	38.59	2.09	2.78	0.05	-0.19	-1.03	35.56	37.53	38.39	40.32	40.99	6
P30000	44.74	1.88	2.82	0.04	0.23	-1.61	42.61	43.21	44.65	46.03	47.33	6
P40000	58.27	5.41	10.03	0.09	0.22	-1.77	51.39	53.83	57.90	63.86	65.19	9

M: Mean, SD: Standard Deviation, IQR: Inter-quartile Range, VC:Variation Coefficient, Sk: Skewness, K: Kurtosis, DN: Data number.

STATISTICAL ANALYSIS.

PART II: Activity of the Biospeckle Pattern as a function of the concentration of benznidazole

DATA. The following data correspond to the benznidazole experiment (Fig 12), with and without parasites and increasing concentrations of the drug, with the addition in each case, of the value for zero concentration. The data were organized in two groups, Group 1 (with parasites and benznidazole) and Group 2 (without parasites and with benznidazole). Each group was further divided in two subgroups: 1,1 with parasites and low concentrations of the drug (0 to 1.25μg/mL); 1,2 with parasites and high concentrations of the drug (5 and 20μg/mL); 2,1 without parasites and low concentrations of the drug (0 to 1.25μg/mL); 2,2 without parasites and high concentrations of the drug (5 and 20μg/mL).

Concentration	Name	C	Group	Subgroup
20	Parasites in LIT t1min	42.312	GROUP 1	GROUP 1,2
5	Parasites in LIT t1min	40.17	GROUP 1	GROUP 1,2
1.25	Parasites in LIT t1min	44.36	GROUP 1	GROUP 1,1
0.3125	Parasites in LIT t1min	45.175	GROUP 1	GROUP 1,1
0.078125	Parasites in LIT t1min	48.32	GROUP 1	GROUP 1,1
0.01953125	Parasites in LIT t1min	48.568	GROUP 1	GROUP 1,1
0	Parasites in LIT t1min	53.504	GROUP 1	GROUP 1,1
20	Parasites in LIT t61min	41.52	GROUP 1	GROUP 1,2
5	Parasites in LIT t61min	41.051	GROUP 1	GROUP 1,2
1.25	Parasites in LIT t61min	45.36	GROUP 1	GROUP 1,1
0.3125	Parasites in LIT t61min	48.3	GROUP 1	GROUP 1,1
0.078125	Parasites in LIT t61min	47.795	GROUP 1	GROUP 1,1
0.01953125	Parasites in LIT t61min	49.569	GROUP 1	GROUP 1,1
0	Parasites in LIT t61min	53.319	GROUP 1	GROUP 1,1
20	Parasites in LIT t128min	46.37	GROUP 1	GROUP 1,2
5	Parasites in LIT t128min	44.971	GROUP 1	GROUP 1,2
1.25	Parasites in LIT t128min	44.94	GROUP 1	GROUP 1,1
0.3125	Parasites in LIT t128min	44.569	GROUP 1	GROUP 1,1
0.078125	Parasites in LIT t128min	47.231	GROUP 1	GROUP 1,1
0.01953125	Parasites in LIT t128min	47.549	GROUP 1	GROUP 1,1
0	Parasites in LIT t128min	52.742	GROUP 1	GROUP 1,1
20	Parasites in LIT t204min	30.59	GROUP 1	GROUP 1,2
5	Parasites in LIT t204min	33.945	GROUP 1	GROUP 1,2
1.25	Parasites in LIT t204min	46.419	GROUP 1	GROUP 1,1
0.3125	Parasites in LIT t204min	48.741	GROUP 1	GROUP 1,1
0.078125	Parasites in LIT t204min	48.727	GROUP 1	GROUP 1,1
0.01953125	Parasites in LIT t204min	49.78	GROUP 1	GROUP 1,1
0	Parasites in LIT t204min	52.763	GROUP 1	GROUP 1,1
20	LIT t1min	28.106	GROUP 2	GROUP 2,2
5	LIT t1min	24.848	GROUP 2	GROUP 2,2

1.25	LIT t1min	23.864	GROUP 2	GROUP 2,1
0.3125	LIT t1min	20.259	GROUP 2	GROUP 2,1
0.01953125	LIT t1min	27.207	GROUP 2	GROUP 2,1
0	LIT t1min	24.996	GROUP 2	GROUP 2,1
20	LIT t61min	22.64	GROUP 2	GROUP 2,2
5	LIT t61min	22.64	GROUP 2	GROUP 2,2
1.25	LIT t61min	17.92	GROUP 2	GROUP 2,1
0.3125	LIT t61min	13.53	GROUP 2	GROUP 2,1
0.01953125	LIT t61min	26.97	GROUP 2	GROUP 2,1
0	LIT t61min	26.2	GROUP 2	GROUP 2,1
20	LIT t128min	23.203	GROUP 2	GROUP 2,2
5	LIT t128min	18.86	GROUP 2	GROUP 2,2
1,25	LIT t128min	17.634	GROUP 2	GROUP 2,1
0,3125	LIT t128min	17.972	GROUP 2	GROUP 2,1
0,01953125	LIT t128min	28.014	GROUP 2	GROUP 2,1
0	LIT t128min	23.574	GROUP 2	GROUP 2,1
20	LIT t204min	22.163	GROUP 2	GROUP 2,2
5	LIT t204min	23.812	GROUP 2	GROUP 2,2
1.25	LIT t204min	22.258	GROUP 2	GROUP 2,1
0.3125	LIT t204min	21.498	GROUP 2	GROUP 2,1
0.01953125	LIT t204min	29.305	GROUP 2	GROUP 2,1
0	LIT t204min	18.757	GROUP 2	GROUP 2,1

R commander denomination: statistics/numerical summary

	mean	sd	data:n
GROUP 1	46.02357	5.272927	28
GROUP 2	22.75958	3.947684	24

	mean	sd	data:n
GROUP 1,1	48.38655	2.907924	20
GROUP 1,2	40.11612	5.334259	8
GROUP 2,1	22.49738	4.527660	16
GROUP 2,2	23.28400	2.608804	8

ANOVA MODEL

NULL HYPOTHESIS: The Means for Groups 1,1; 1,2; 2,1 and 2,2 are equal.

ALTERNATIVE HYPOTHESIS: At least the mean of one of the four Groups is statistically different.

	Df	Sum Sq	Mean Sq	F value	Pr(>F)
Subgroup	3	7388	2462.8	165.3	<2e-16 ***
Residuals	48	715	14.9		

Signif. codes: 0 '***' 0.001 '**' 0.01 '*' 0.05 '.' 0.1 ' ' 1

```
> numSummary(Datos$C , groups=Datos$Subgrupo, statistics=c("mean", "sd"))
```

	mean	sd	data:n
GROUP 1,1	48.38655	2.907924	20
GROUP 1,2	40.11612	5.334259	8
GROUP 2,1	22.49738	4.527660	16
GROUP 2,2	23.28400	2.608804	8

Since the the p-value ($2x10^{-16}$) is less than the significance level of 0.05, the null hypothesis stating that the mean of the four Groups is equal, is rejected.

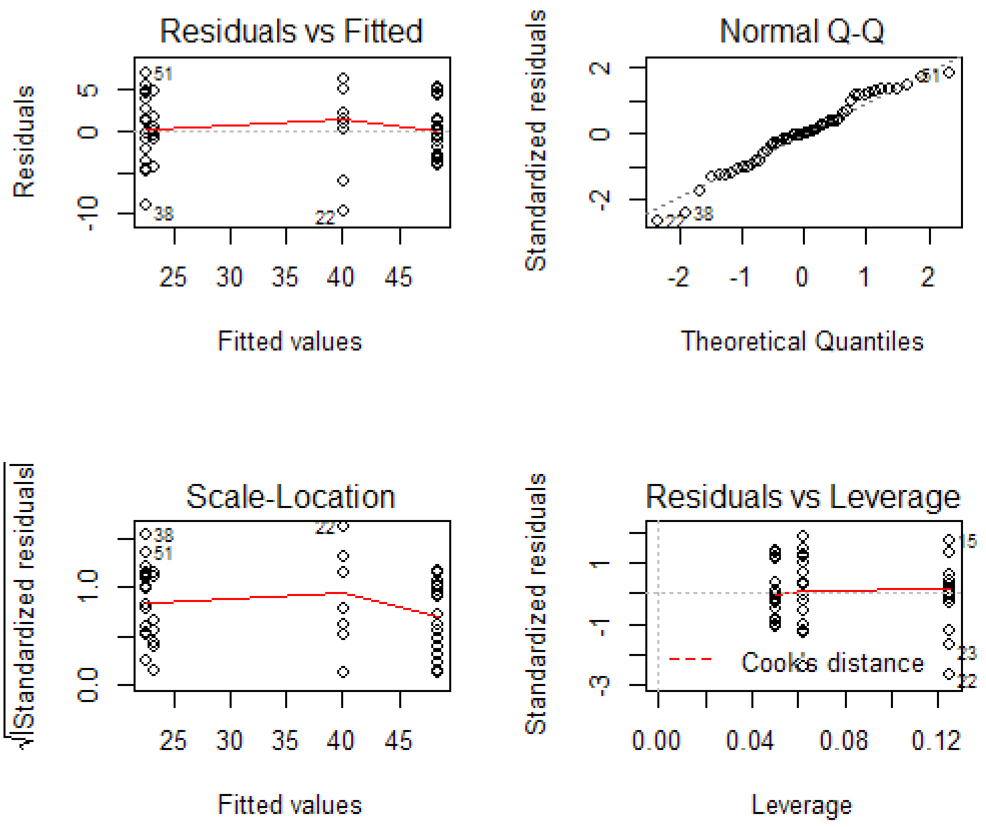

Residuals of the ANOVA Model

TUKEY POST HOC

Since the Null Hypothesis was rejected, a Tukey *post hoc* Test was performed in order to find the combinations by pairs that have statistically different means. The p-values for 5 of the combinations are less than the significance level of 0.05 which indicates that the mean value between pairs, are statistically different. Only in the case of **GROUP 2,2-GROUP 2,1** the p-value is higher than the significance level of 0.05 which indicates that the mean value between pairs are statistically equal.

Tukey multiple comparisons of means
95% family-wise confidence level

Fit: aov(formula = C ~ Subgroup, data = Datos)

$Subgroup

	diff	lwr	upr	p adj
GROUP 1,2-GROUP 1,1	-8.270425	-12.567287	-3.973563	0.0000308
GROUP 2,1-GROUP 1,1	-25.889175	-29.334329	-22.444021	0.0000000
GROUP 2,2-GROUP 1,1	-25.102550	-29.399412	-20.805688	0.0000000
GROUP 2,1-GROUP 1,2	-17.618750	-22.066424	-13.171076	0.0000000
GROUP 2,2-GROUP 1,2	-16.832125	-21.967857	-11.696393	0.0000000
GROUP 2,2-GROUP 2,1	0.786625	-3.661049	5.234299	0.9651403

Bartlett test of homogeneity of variances

data: C by Subgroup

Bartlett's K-squared = 6.6359, df = 3, p-value = 0.08445

Since the p-value (0.08445) is higher than 0.05, the Null Hypothesis is accepted, indicating that the variances are statistically equal. In other words, the variability of the four groups is statistically equal.

Levene's Test for Homogeneity of Variance (center = median)

	Df	F value	Pr(>F)
group	3	2.1949	0.1008
	48		

Since the p-value (0.1008) is higher than 0.05, the Null Hypothesis is accepted, indicating that the variances are statistically equal. Again, the variability of the four groups is statistically equal.
