# Supplementary material for: Quantitative Laser Biospeckle Method for the Evaluation of the Activity of Trypanosoma cruzi Using VDRL Plates and Digital Analysis
Source: PLoS Negl Trop Dis. 2016 Dec 5;10(12):e0005169. doi: 10.1371/journal.pntd.0005169 (PMC5137869; doi:10.1371/journal.pntd.0005169)
Supplement: S1 Text — User’s Manual for the Program Image Delta Processor (ImageDP) designed and written in Java for this work. (PDF) [file pntd.0005169.s002.pdf]

S1 Text

Documentation ImageDP

QUANTITATIVE LASER BIOSPECKLE METHOD FOR THE EVALUATION OF THE ACTIVITY OF *Trypanosoma cruzi* USING VDRL PLATES AND DIGITAL ANALYSIS

Hilda Cristina Grassi, Lisbette C. García, María Lorena Lobo-Sulbarán, Ana Velásquez, Francisco A. Andrades-Grassi, Humberto Cabrera, Jesús E. Andrades-Grassi, Efrén D.J. Andrades

Image Delta Processor (ImageDP)

```
/*
* Processes one folder and outputs one result. The folder must contain only image
files.
* One grand total is output at the end. Please see the inline comments for more
information on
* the used algorithm.
*
* Generally, if 3 images are present in the folder with the following grayscale pixel
values:
*
* Image 1 (i1):
*
* | 1 3 1 |
* | 0 1 2 |
* | 2 2 1 |
*
* Image 2 (i2):
*
* | 2 3 1 |
* | 1 1 3 |
* | 2 4 1 |
*
* Image 3 (i3):
*
* | 2 2 0 |
* | 1 1 3 |
* | 1 4 0 |
*
* The images will first be converted to one dimensional arrays:
*
* i1: [1, 2, 1, 0, 1, 2, 2, 2, 1]
* i2: [2, 3, 1, 1, 1, 3, 2, 4, 1]
* i3: [2, 2, 0, 1, 1, 3, 1, 4, 0]
*
* The averages will be calculated by doing a pixel by pixel difference calculation
using:
*
* (abs(i1[0] - i2[0]) + abs(i1[1] - i2[1]) + ... + abs(i1[n] - i2[n])) / n
*
* where n is the image width*height. So the averages in this example will be:
*
* avg(i1, i2): (1 + 1 + 0 + 1 + 0 + 1 + 0 + 2 + 0) / 9 = 0.667
* avg(i2, i3): (0 + 1 + 1 + 0 + 0 + 0 + 1 + 0 + 1) / 9 = 0.444
*
* The average of the two averages is:
*
* avg(avg(i1, i2), avg(i2, i3)): (0.667 + 0.444) / 2 = 0.5556
*
```

```
* So 0.5556 is the result for the folder being processed.
*/
```

The algorithm is:

```
1.- List all files in the folder
2.- Sort the files using the number in their name.
3.- Global counters:
3.1.- tot will store the sum of all the averages
3.2.- count will store the number of averages
4.- Iterate through the sorted list of files
4.1.- file1 is the image file at the current iterator position
4.2.- file2 is the next image file
4.3.- Load both images into memory. This step loads the actual image file.
4.4.- Extract the pixel array of image values from both images. This is a one
dimensional array
with length w*h, where w is the image width and h is the image height. We will now
process the
array linearly, as the process is a per pixel comparison, not a two dimensional
operation.
Please note that the images are assumed to be in grayscale, as we cannot extract a
single numeric
value for comparison and average. Only the R channel is stored. Please see the
getImagePixels
documentation for more information.
4.5.- sum will store the sum of all the pixel differences between image1 and image2
4.6.- Iterate through all the pixels in image1
4.6.1.- Calculate the absolute value of the difference between the pixel in image1 and
image2
at the same index (j)
4.6.2.- Add the difference to the sum
4.7.- Calculate the average, which is the sum of differences divided by the length of
the one dimensional
array containing the image pixels
4.8.- Increase the total sum of averages
4.9.- Increase the counter of number of averages
5.- Calculate the final average, which is the total averages divided by the number of
comparisons made.
6.- Display the result
```

The documentation of the function that extracts the pixels from the images is:

```
/*
* Extracts the pixel values from the image that was read from disk. Please note that
RGB images will not
* be correctly processed, as we cannot extract one single value per pixel (each pixel
contains three values:
* R, G and B). Since images are assumed grayscale before processing, we will only
store the R channel in the
* final array.
*
* The final array is stored in only one dimension, as we will process the images
linearly (we will do a pixel
* by pixel comparison of two images calculating the difference).
*/
```
