# Supplementary material for: Quantitative Laser Biospeckle Method for the Evaluation of the Activity of Trypanosoma cruzi Using VDRL Plates and Digital Analysis
Source: PLoS Negl Trop Dis. 2016 Dec 5;10(12):e0005169. doi: 10.1371/journal.pntd.0005169 (PMC5137869; doi:10.1371/journal.pntd.0005169)
Supplement: S2 Text — Source code for the Program Image Delta Processor (ImageDP). (PDF) [file pntd.0005169.s003.pdf]

S2 Text

Source Image DP

QUANTITATIVE LASER BIOSPECKLE METHOD FOR THE EVALUATION OF THE ACTIVITY OF *Trypanosoma cruzi* USING VDRL PLATES AND DIGITAL ANALYSIS

Hilda Cristina Grassi, Lisbette C. García, María Lorena Lobo-Sulbarán, Ana Velásquez, Francisco A. Andrades-Grassi, Humberto Cabrera, Jesús E. Andrades-Grassi, Efrén D.J. Andrades

Image Delta Processor (ImageDP)

```
package com.bigjocker.images;

import java.awt.image.BufferedImage;
import java.awt.image.PixelGrabber;
import java.io.File;
import java.util.Arrays;
import java.util.Comparator;
import java.util.StringTokenizer;

import javax.imageio.ImageIO;

public class ImagesProcessor {
	public static void main(String[] args) throws Exception {
		for (int i = 0 ; i < args.length ; i++) {
			ImagesProcessor.processAll(args[i]);
		}
	}

	public static void processAll(String rootFolder) throws Exception {
		File root = new File(rootFolder);
		File[] allFiles = root.listFiles();
		for (File dir: allFiles) {
			if (dir.isDirectory()) {
				ImagesProcessor.process(dir.getAbsolutePath());
			}
		}
	}

	public static void process(String folder) throws Exception {
		File root = new File(folder);
		File[] allFiles = root.listFiles();

		Arrays.sort(allFiles, new Comparator<File>() {
			public int compare(File s1, File s2) {
				StringTokenizer st1 = new StringTokenizer(s1.getName(), " ");
				StringTokenizer st2 = new StringTokenizer(s2.getName(), " ");
```

```
					while (st1.hasMoreTokens() && st2.hasMoreTokens()) {
						String t1 = st1.nextToken();
						String t2 = st2.nextToken();

						int c;
						try {
							Integer i1 = new Integer(t1);
							Integer i2 = new Integer(t2);
							c = i1.compareTo(i2);
						} catch (NumberFormatException e) {
							c = t1.compareTo(t2);
						}
						if (c != 0) {
							return c;
						}
					}

					return 0;
				}
			});

			float tot = 0;
			int count = 0;
			int percents[] = new int[100];
			System.out.print("  ");
			for (int i = 0 ; i < allFiles.length - 1 ; i++) {
				File file1 = allFiles[i];
				File file2 = allFiles[i + 1];

				BufferedImage img1 = ImageIO.read(file1);
				BufferedImage img2 = ImageIO.read(file2);

				int[] m1 = ImagesProcessor.getImagePixels(img1);
				int[] m2 = ImagesProcessor.getImagePixels(img2);

				long sum = 0;
				for (int j = 0 ; j < m1.length ; j++) {
					int val = Math.abs(m2[j] - m1[j]);
					sum += val;
				}
				float avg = ((float) sum) / m1.length;

				tot += avg;
				count++;

				int percent = count*100/allFiles.length;
				if (percents[percent] == 0) {
					System.out.print("\b\b");
					if (percent >= 10) {
```

```
                        System.out.print("\b");
                    }
                    System.out.print(percent + "%");
                    percents[percent] = 1;
                }
            }

            float totavg = tot/count;
            System.out.print("\b\b\b");
            System.out.println("For '" + root.getAbsolutePath() + "' / Total average: " + totavg);
        }

        public static int[] getImagePixels(BufferedImage image) {
            PixelGrabber grabber = null;
            int[] pixels = new int[image.getWidth() * image.getHeight()];
            try {
                grabber = new PixelGrabber(image, 0, 0, image.getWidth(), image.getHeight(),
pixels, 0, image.getWidth());
                grabber.grabPixels(0);

                for (int i = 0 ; i < pixels.length ; i++) {
                    int pixel = pixels[i];

                    int alpha = (pixel >> 24) & 0xff;
                int red   = (pixel >> 16) & 0xff;
                int green = (pixel >>  8) & 0xff;
                int blue  = (pixel      ) & 0xff;

                pixels[i] = red;
                }
            } catch (Exception e) {
                e.printStackTrace();
            }
            return pixels;
        }
}
```
