# Supplementary material for: Quantitative Laser Biospeckle Method for the Evaluation of the Activity of Trypanosoma cruzi Using VDRL Plates and Digital Analysis
Source: PLoS Negl Trop Dis. 2016 Dec 5;10(12):e0005169. doi: 10.1371/journal.pntd.0005169 (PMC5137869; doi:10.1371/journal.pntd.0005169)
Supplement: S3 Text — Settings for Program Image Delta Processor (ImageDP). (PDF) [file pntd.0005169.s004.pdf]

S3 Text

Settings Image DP

QUANTITATIVE LASER BIOSPECKLE METHOD FOR THE EVALUATION OF THE ACTIVITY OF *Trypanosoma cruzi* USING VDRL PLATES AND DIGITAL ANALYSIS

Hilda Cristina Grassi, Lisbette C. García, María Lorena Lobo-Sulbarán, Ana Velásquez, Francisco A. Andrades-Grassi, Humberto Cabrera, Jesús E. Andrades-Grassi, Efrén D.J. Andrades

Image Delta Processor (ImageDP)

```
eclipse.preferences.version=1
org.eclipse.jdt.core.compiler.codegen.inlineJsrBytecode=enabled
org.eclipse.jdt.core.compiler.codegen.targetPlatform=1.6
org.eclipse.jdt.core.compiler.codegen.unusedLocal=preserve
org.eclipse.jdt.core.compiler.compliance=1.6
org.eclipse.jdt.core.compiler.debug.lineNumber=generate
org.eclipse.jdt.core.compiler.debug.localVariable=generate
org.eclipse.jdt.core.compiler.debug.sourceFile=generate
org.eclipse.jdt.core.compiler.problem.assertIdentifier=error
org.eclipse.jdt.core.compiler.problem.enumIdentifier=error
org.eclipse.jdt.core.compiler.source=1.6
```
