# Supplementary material for: Quantitative Laser Biospeckle Method for the Evaluation of the Activity of Trypanosoma cruzi Using VDRL Plates and Digital Analysis
Source: PLoS Negl Trop Dis. 2016 Dec 5;10(12):e0005169. doi: 10.1371/journal.pntd.0005169 (PMC5137869; doi:10.1371/journal.pntd.0005169)
Supplement: S4 Text — Project for Image Delta Processor (ImageDP). (PDF) [file pntd.0005169.s005.pdf]

S4 Text

Project Image DP

QUANTITATIVE LASER BIOSPECKLE METHOD FOR THE EVALUATION OF THE ACTIVITY OF *Trypanosoma cruzi* USING VDRL PLATES AND DIGITAL ANALYSIS

Hilda Cristina Grassi, Lisbette C. García, María Lorena Lobo-Sulbarán, Ana Velásquez, Francisco A. Andrades-Grassi, Humberto Cabrera, Jesús E. Andrades-Grassi, Efrén D.J. Andrades

Image Delta Processor (ImageDP)


```
 <?xml version="1.0" encoding="UTF-8" ?>
- <projectDescription>
  <name>ImagesDeltaProcessor</name>
  <comment />
  <projects />
- <buildSpec>
- <buildCommand>
  <name>org.eclipse.jdt.core.javabuilder</name>
  <arguments />
    </buildCommand>
    </buildSpec>
- <natures>
  <nature>org.eclipse.jdt.core.javanature</nature>
    </natures>
    </projectDescription>
```
