# Supplementary material for: Quantitative Laser Biospeckle Method for the Evaluation of the Activity of Trypanosoma cruzi Using VDRL Plates and Digital Analysis
Source: PLoS Negl Trop Dis. 2016 Dec 5;10(12):e0005169. doi: 10.1371/journal.pntd.0005169 (PMC5137869; doi:10.1371/journal.pntd.0005169)
Supplement: S5 Text — Classpath for Image Delta Processor (ImageDP). (PDF) [file pntd.0005169.s006.pdf]

S5 Text

Classpath Image DP

QUANTITATIVE LASER BIOSPECKLE METHOD FOR THE EVALUATION OF THE ACTIVITY OF *Trypanosoma cruzi* USING VDRL PLATES AND DIGITAL ANALYSIS

Hilda Cristina Grassi, Lisbette C. García, María Lorena Lobo-Sulbarán, Ana Velásquez, Francisco A. Andrades-Grassi, Humberto Cabrera, Jesús E. Andrades-Grassi, Efrén D.J. Andrades

Image Delta Processor (ImageDP)


```
<?xml version="1.0" encoding="UTF-8" ?>
- <classpath>
  <classpathentry kind="src" path="src" />
  <classpathentry kind="con"
    path="org.eclipse.jdt.launching.JRE_CONTAINER/org.eclipse.jdt.internal.debu
    g.ui.launcher.StandardVMType/JavaSE-1.6" />
  <classpathentry kind="output" path="bin" />
  </classpath>
```
