# Supplementary material for: Quantitative Laser Biospeckle Method for the Evaluation of the Activity of Trypanosoma cruzi Using VDRL Plates and Digital Analysis
Source: PLoS Negl Trop Dis. 2016 Dec 5;10(12):e0005169. doi: 10.1371/journal.pntd.0005169 (PMC5137869; doi:10.1371/journal.pntd.0005169)
Supplement: S6 Text — Script that uses R Commander/RSAGA/SAGA GIS (R/SAGA), designed and written for this work. (PDF) [file pntd.0005169.s007.pdf]

S6 Text

Script R/SAGA

QUANTITATIVE LASER BIOSPECKLE METHOD FOR THE EVALUATION OF THE ACTIVITY OF *Trypanosoma cruzi* USING VDRL PLATES AND DIGITAL ANALYSIS

Hilda Cristina Grassi, Lisbette C. García, María Lorena Lobo-Sulbarán, Ana Velásquez, Francisco A. Andrades-Grassi, Humberto Cabrera, Jesús E. Andrades-Grassi, Efrén D.J. Andrades

S6 Script R/SAGA

R Commander/RSAGA/SAGA GIS/Equation 2

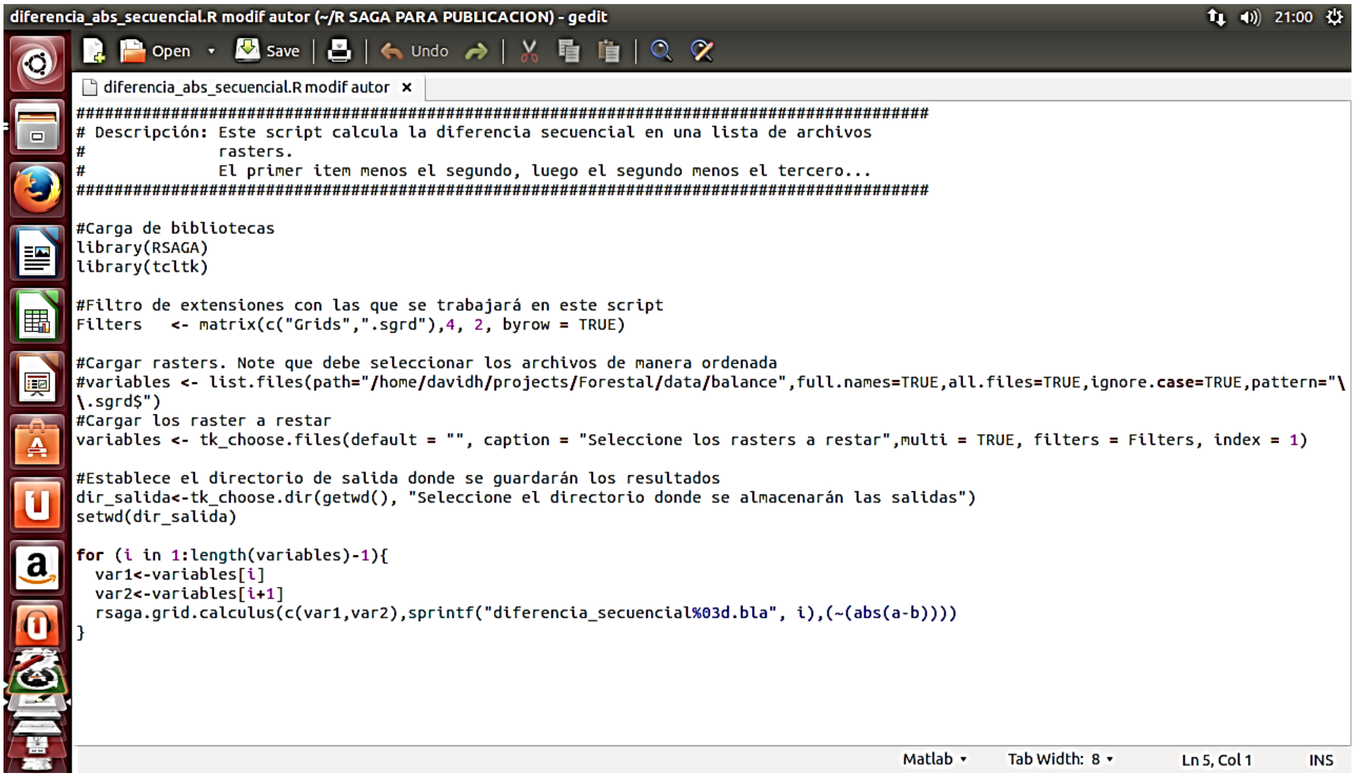


```
##########################################################################################
# Descripción: Este script calcula la diferencia secuencial en una lista de archivos
#              rasters.
#              El primer item menos el segundo, luego el segundo menos el tercero...
##########################################################################################

#Carga de bibliotecas
library(RSAGA)
library(tcltk)

#Filtro de extensiones con las que se trabajará en este script
Filters   <- matrix(c("Grids",".sgrd"),4, 2, byrow = TRUE)

#Cargar rasters. Note que debe seleccionar los archivos de manera ordenada
#variables <- list.files(path="/home/davidh/projects/Forestal/data/balance",full.names=TRUE,all.files=TRUE,ignore.case=TRUE,pattern="\
\.sgrd$")
#Cargar los raster a restar
variables <- tk_choose.files(default = "", caption = "Seleccione los rasters a restar",multi = TRUE, filters = Filters, index = 1)

#Establece el directorio de salida donde se guardarán los resultados
dir_salida<-tk_choose.dir(getwd(), "Seleccione el directorio donde se almacenarán las salidas")
setwd(dir_salida)

for (i in 1:length(variables)-1){
  var1<-variables[i]
  var2<-variables[i+1]
  rsaga.grid.calculus(c(var1,var2),sprintf("diferencia_secuencial%03d.bla", i),(~(abs(a-b))))
}
```
