# Supplementary material for: Quantitative Laser Biospeckle Method for the Evaluation of the Activity of Trypanosoma cruzi Using VDRL Plates and Digital Analysis
Source: PLoS Negl Trop Dis. 2016 Dec 5;10(12):e0005169. doi: 10.1371/journal.pntd.0005169 (PMC5137869; doi:10.1371/journal.pntd.0005169)

S7
M
A
N
U
A
L
R/
S
A
G
A

S7 Text

MANUAL R/SAGA

QUANTITATIVE LASER BIOSPECKLE METHOD FOR THE EVALUATION OF THE ACTIVITY OF *Trypanosoma cruzi* USING VDRL PLATES AND DIGITAL ANALYSIS

Hilda Cristina Grassi, Lisbette C. García, María Lorena Lobo-Sulbarán, Ana Velásquez, Francisco A. Andrades-Grassi, Humberto Cabrera, Jesús E. Andrades-Grassi, Efrén D.J. Andrades

This script was developed by the research group from Venezuela, therefore it was written in Spanish. This User´s Manual is a translation to English, but the script and the version of the program are in Spanish.

INSTALLATION OF R WITH LINUX OPERATING SYSTEM

- Open Ubuntu, make sure you have an internet connection,
- Go to Ubuntu Software Center and install R and Rcmdr, with Optional ad-ons GNU R package for ODBC database access (r-cran-rodbc)
- When you open Rcmdr the following graphic interface will open

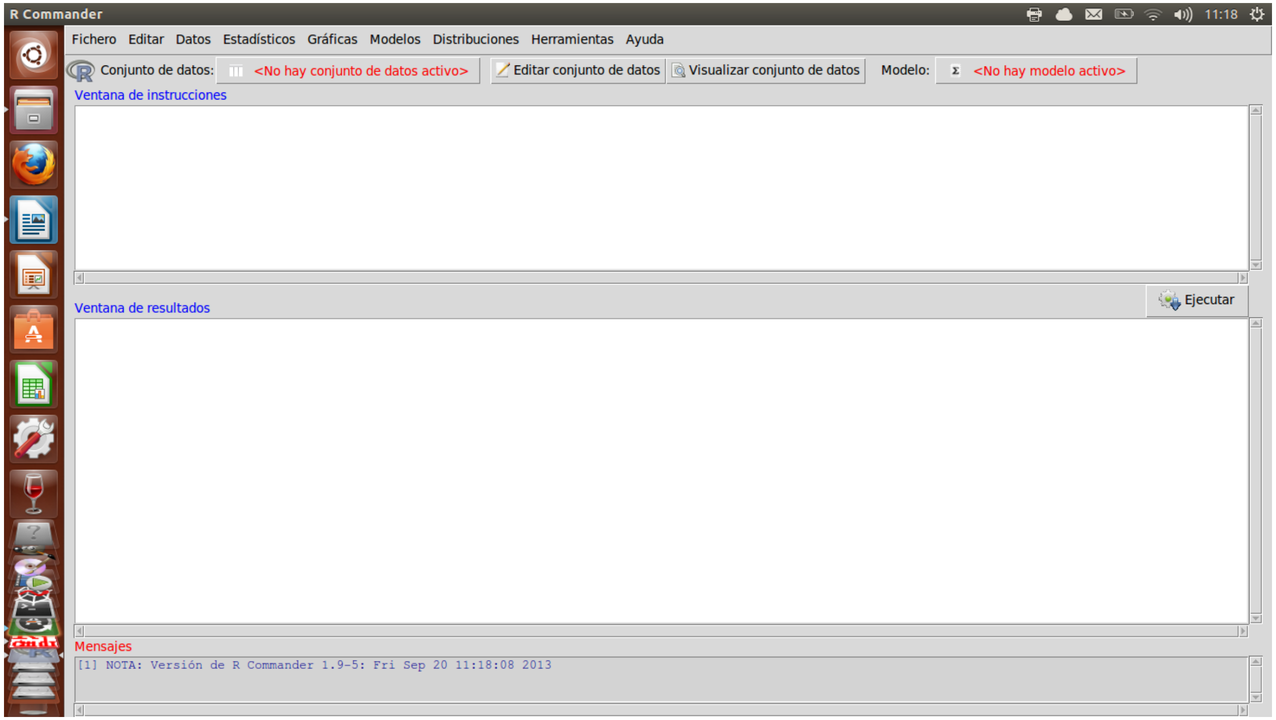

- Type in the console **install.packages ("spatstat"); install.packages ("RSAGA”)** and **install.packages ("tcltk”)** and Execute (**Submit**),
- Select the mirror from which the library will be downloaded, any of the mirrors can be used, **0-cloud** is recommended, click **OK**

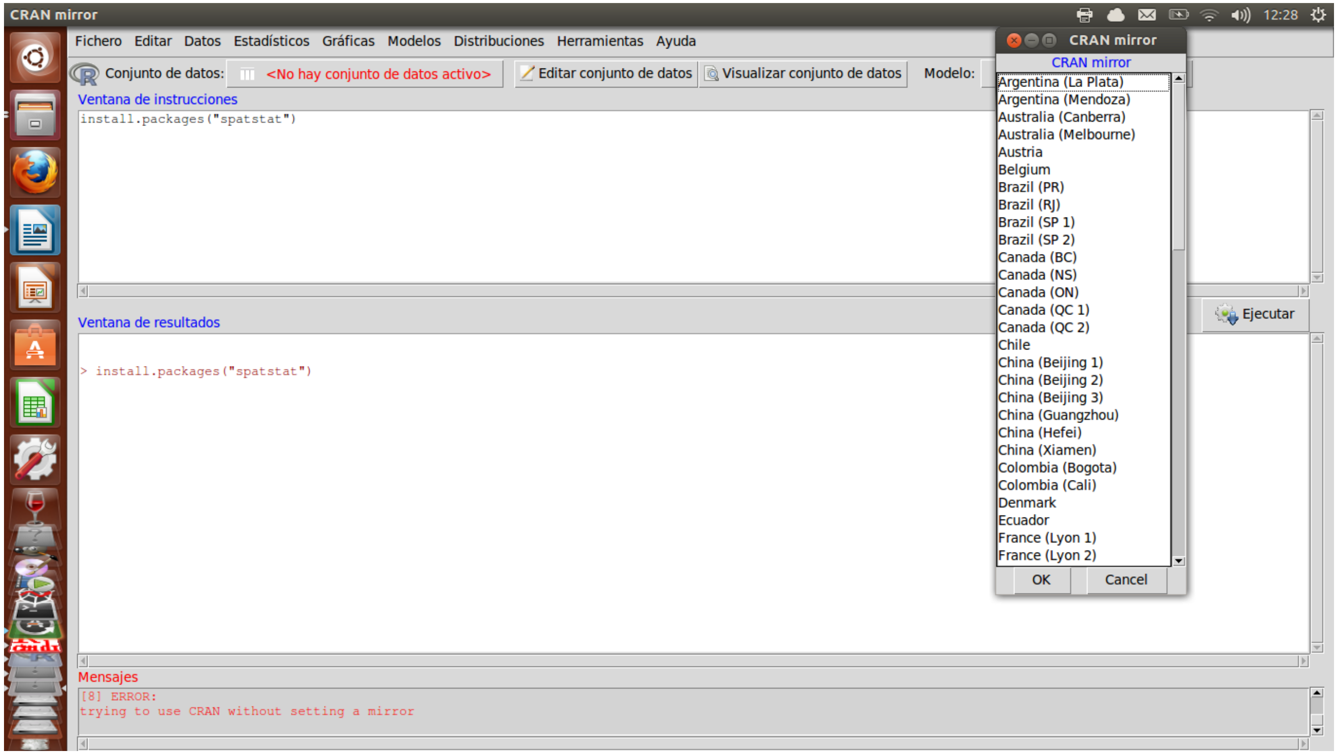

PREPARATION OF THE IMAGE RASTERS

The first step is to **Convert the video to jpg** with the converter of your preference.

The second step prepares the frames as **.sgrd** files that can be recognized by Rcommander.

- **Open SAGA GIS**, close the window **Tip of the Day**
- Go to **Module/Import-Export GDAL/OGR**
- Go to **GDAL Import raster**
- Select the folder where the frames are saved and select the frames that are to be imported.

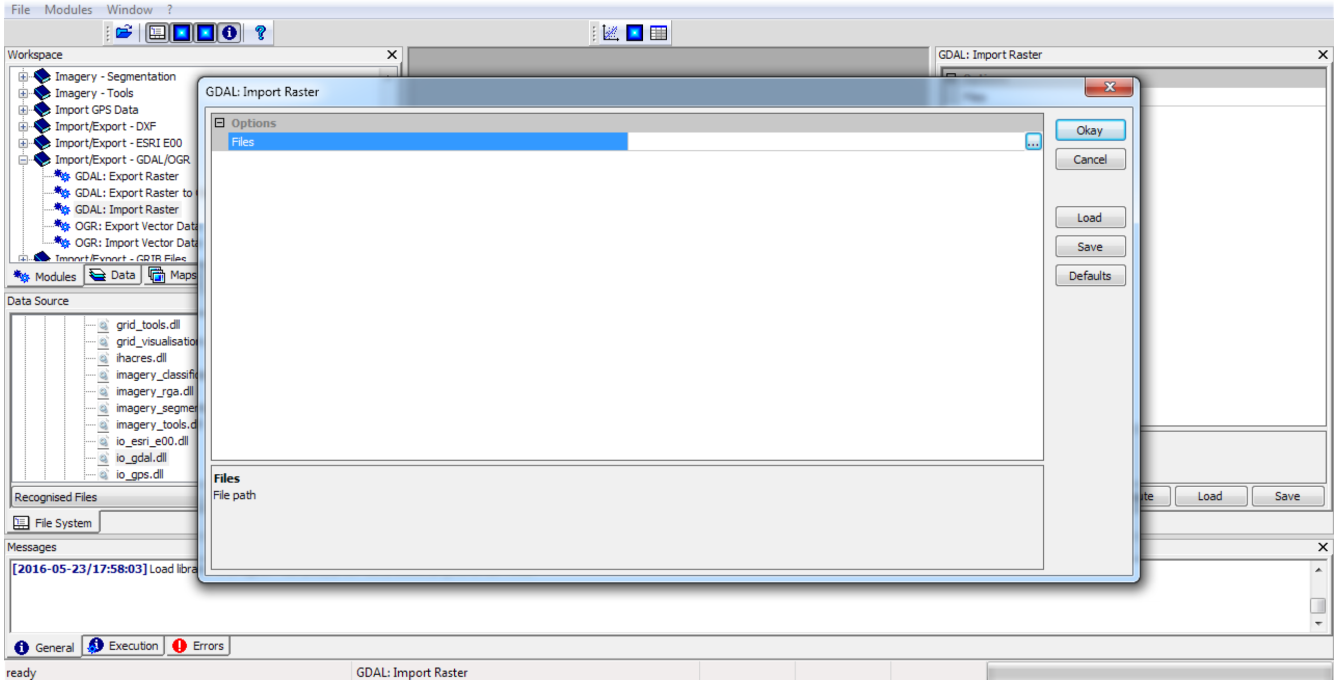

Once the frames are imported,

- Go to **File/Project/Save Project As/** select the folder where the project will be saved

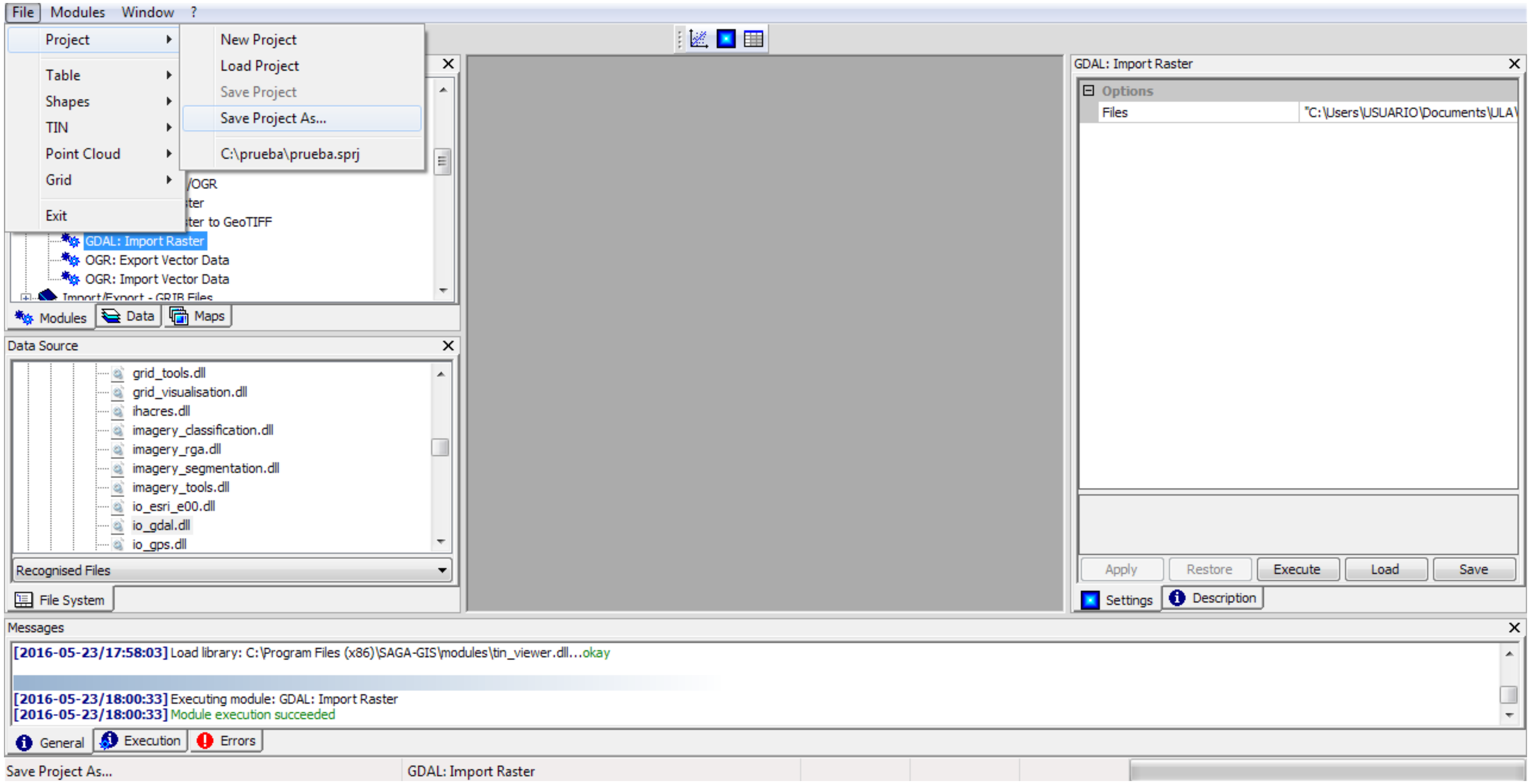

EXECUTION OF THE SCRIPT R/SAGA (abs sequential)

This is a script that subtracts frames that are in a sequence and creates the rasters with the absolute value of the difference. For example, ($frame_2$ – $frame_1$); ($frame_3$ – $frame_2$)….. The product will be a raster which is the absolute value of the difference that will also be in a sequence.

Fort he execution of the script you must have the following installed:

Ubuntu 12.10 or higher

R 3.0 or higher

R commander (Recommended) or Rstudio

- Open Ubuntu,
- Open **R commander (Rcmdr**)

The following graphic interface will open

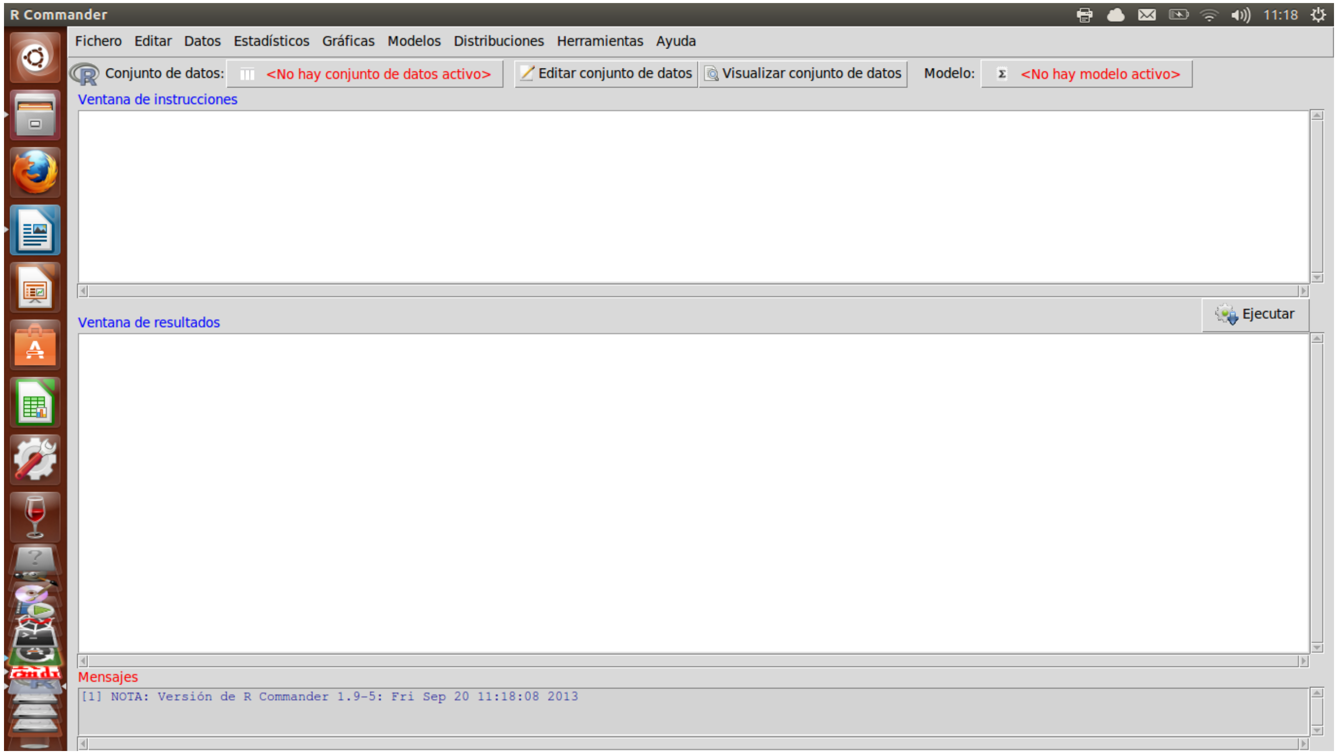

- Go to the menu **File/Open instruction file**

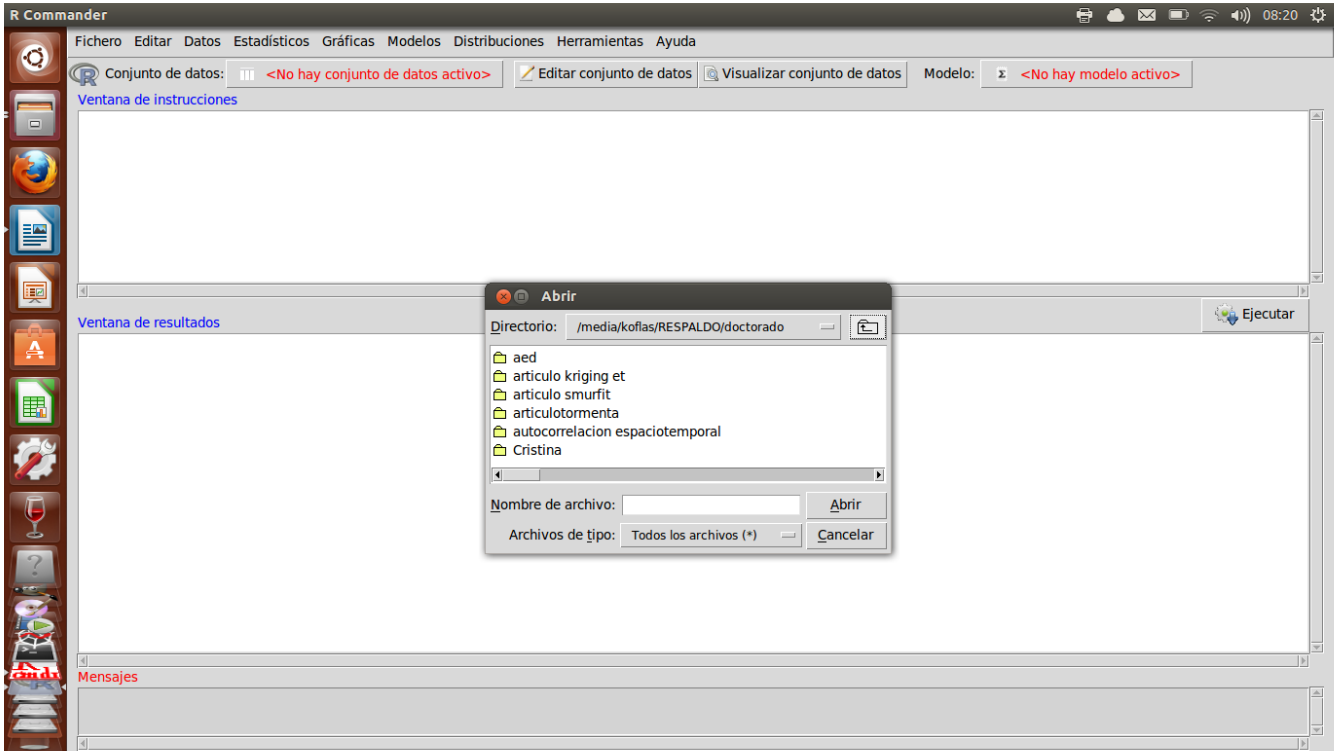

- Go to the location of the script **(diferencia_abs_secuencial.R)** and press **Open**

The script will load a series of instructions (The symbol # defines a commentary in the script)

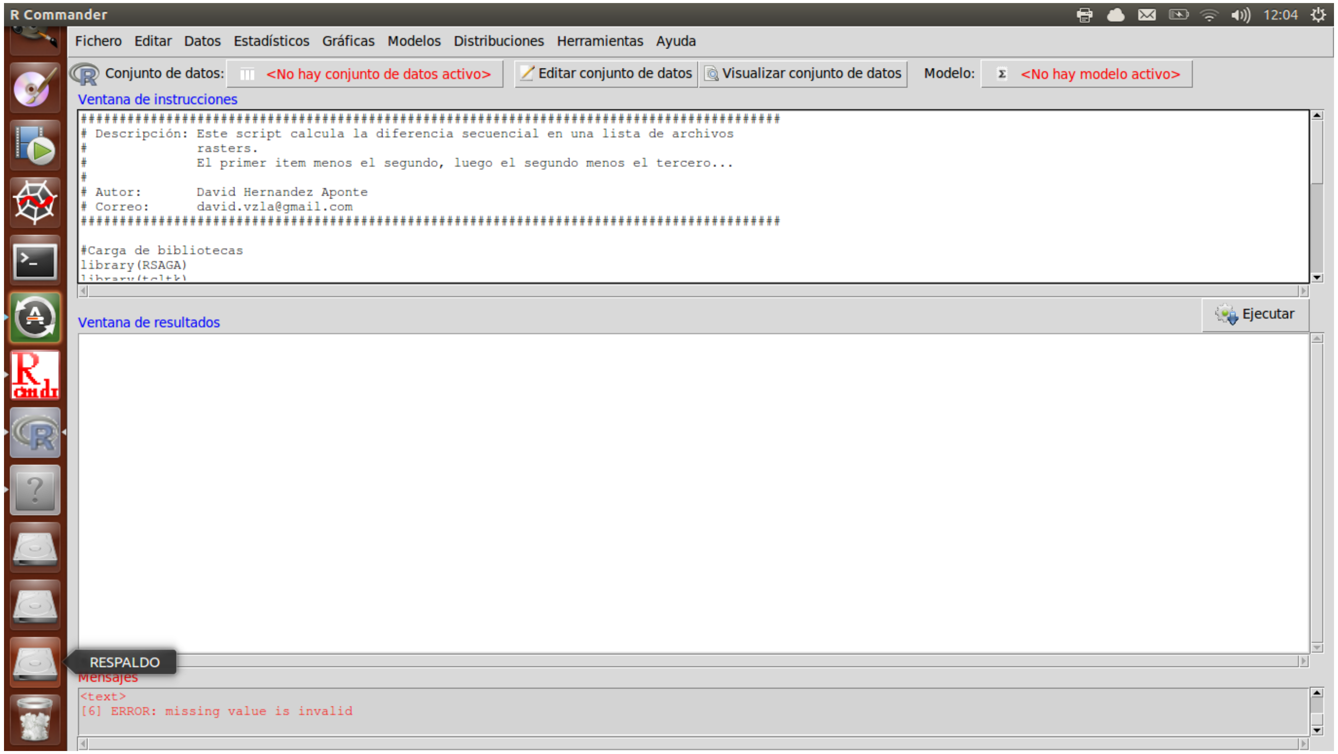

S7 MANUAL R/SAGA

In order to load the RSAGA and tcltk libraries

- Select the code **library(RSAGA) library(tcltk)** and press **Execute (Submit)** which is at the right of the screen of Rcmdr

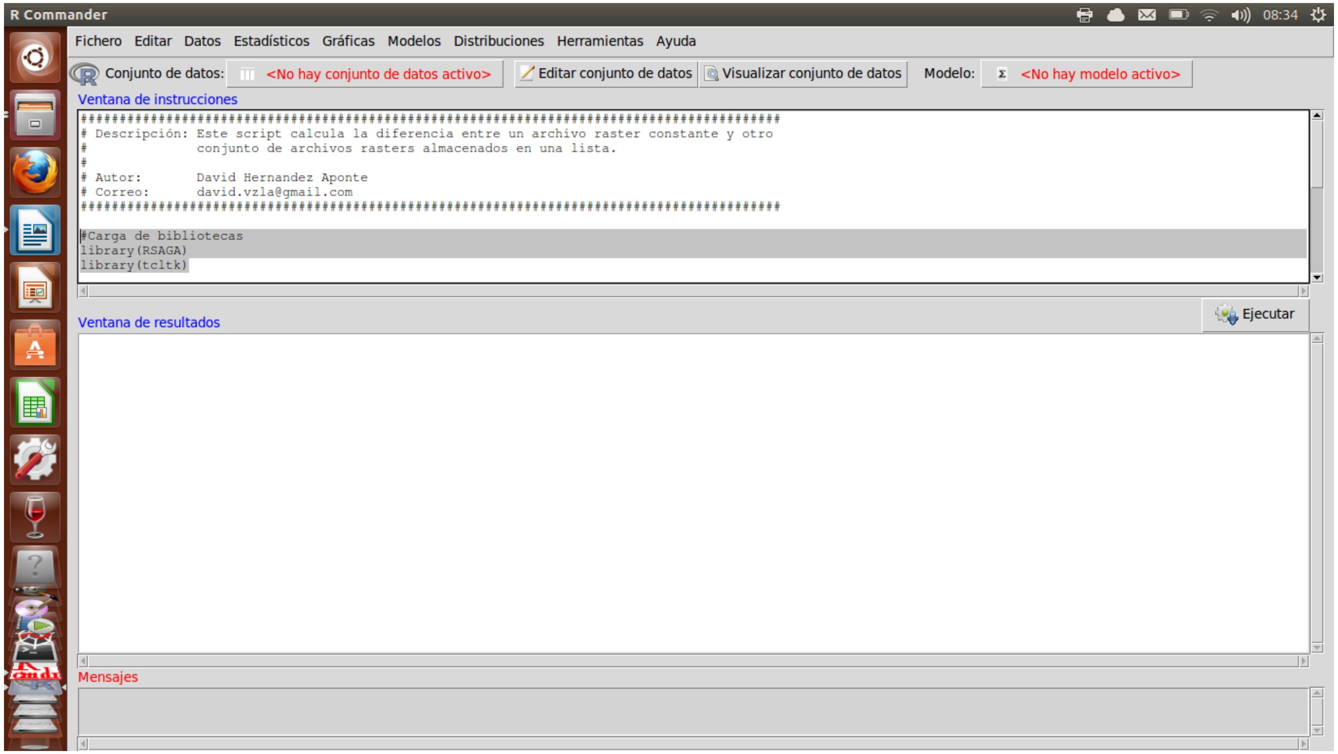

Note that the products of the executed code will appear in the results window

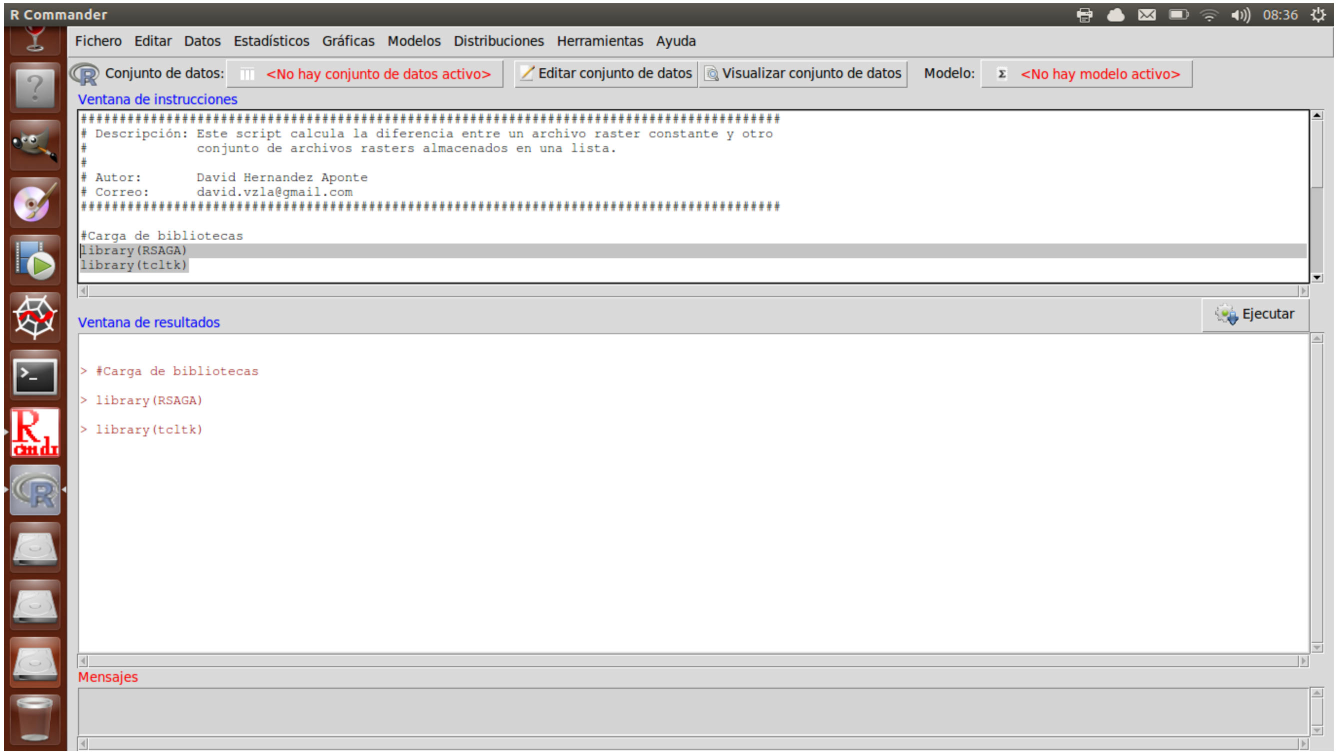

- Move the cursor down and you will note that there is another code to execute.
- Execute the Extensions Filter with which you will work in this script, you will define the extension of the files which will be Saga Grid (.sgrd)

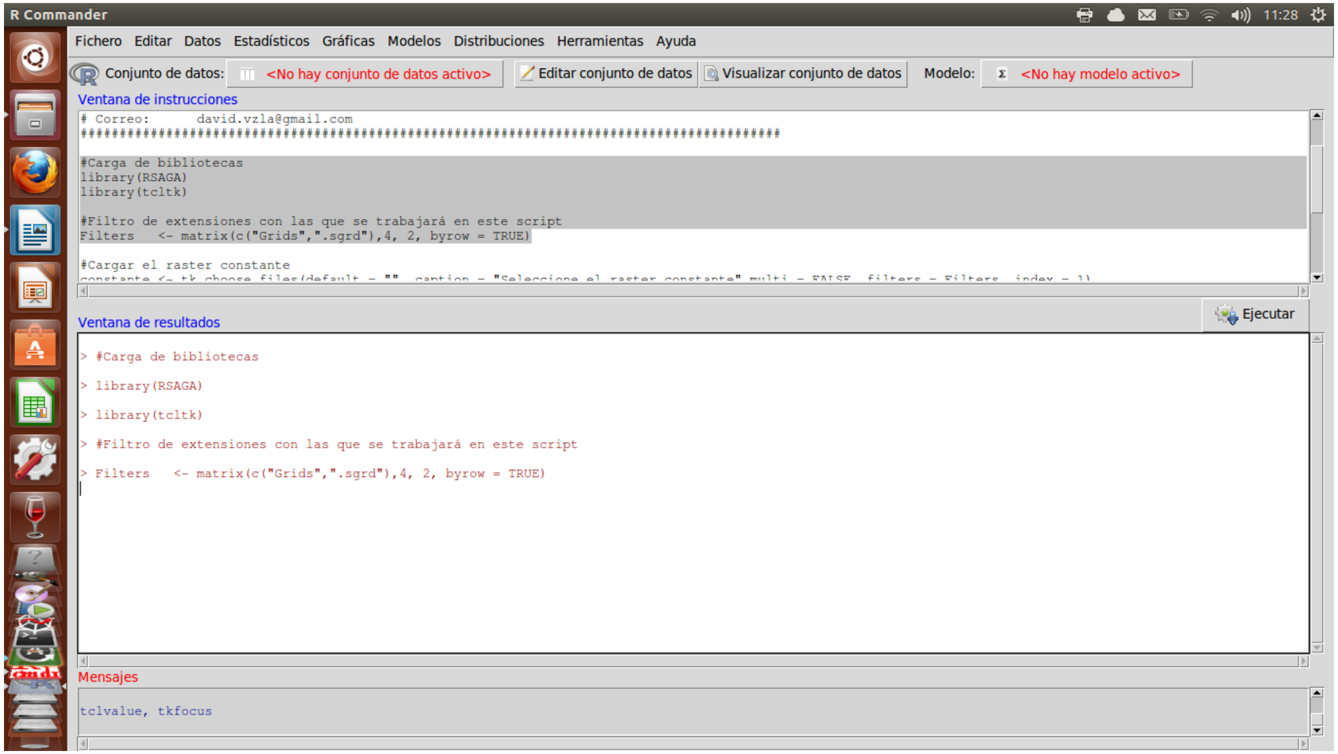

- Execute the instruction to load the rasters

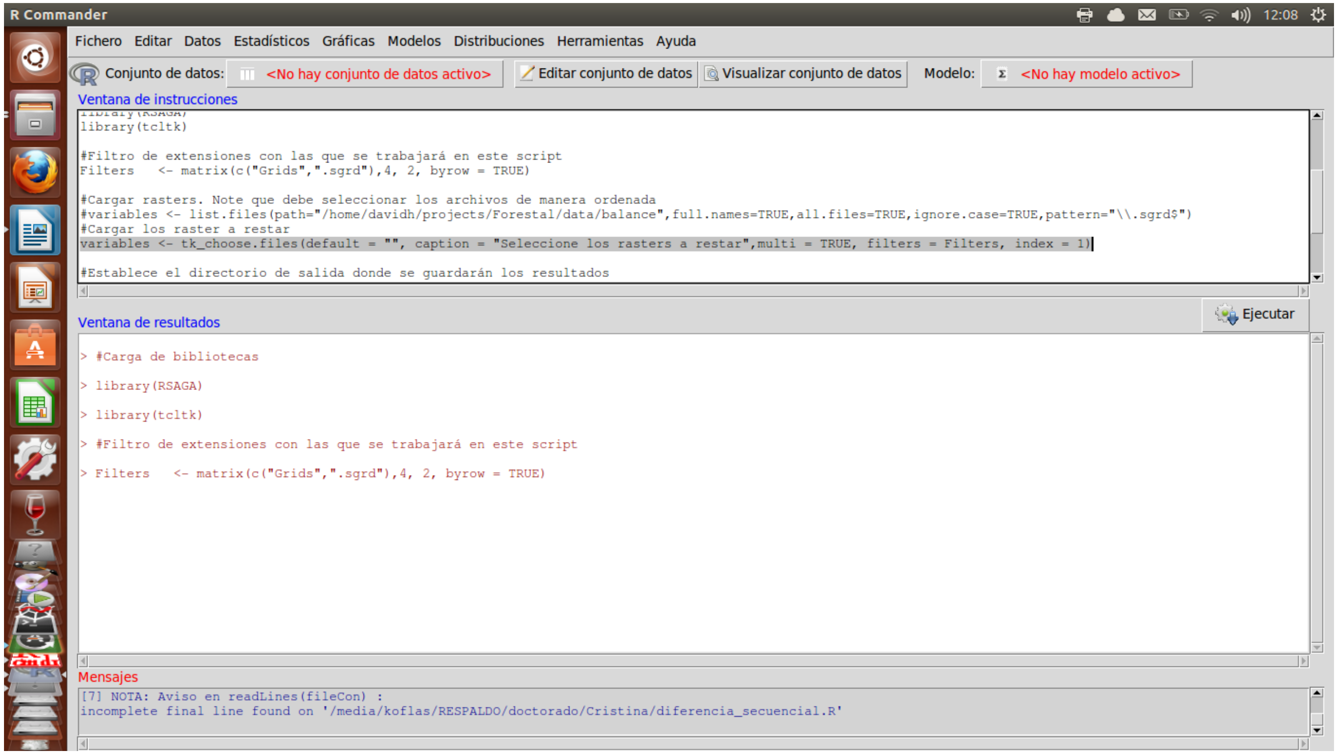

- Load the frames or in an ordered manner

Note that you can load multiple files

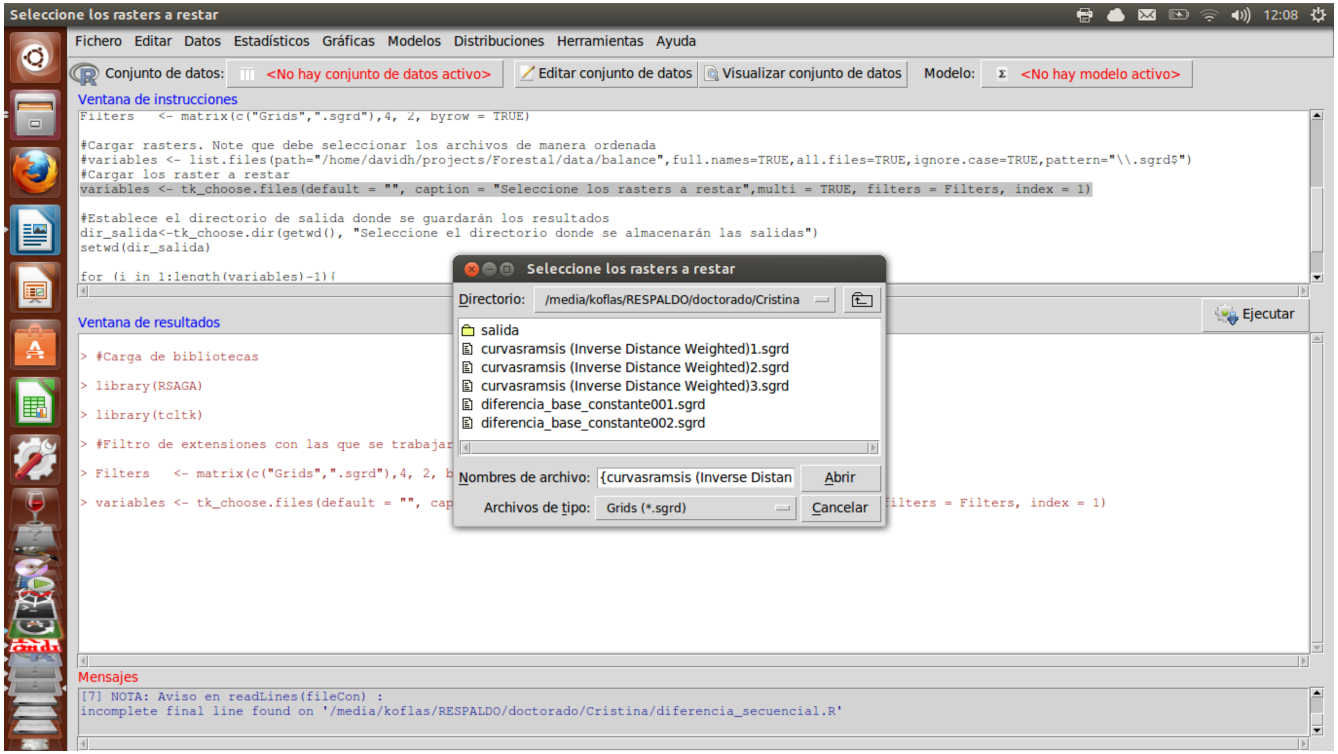

- Choose the output file where the difference rasters will be saved

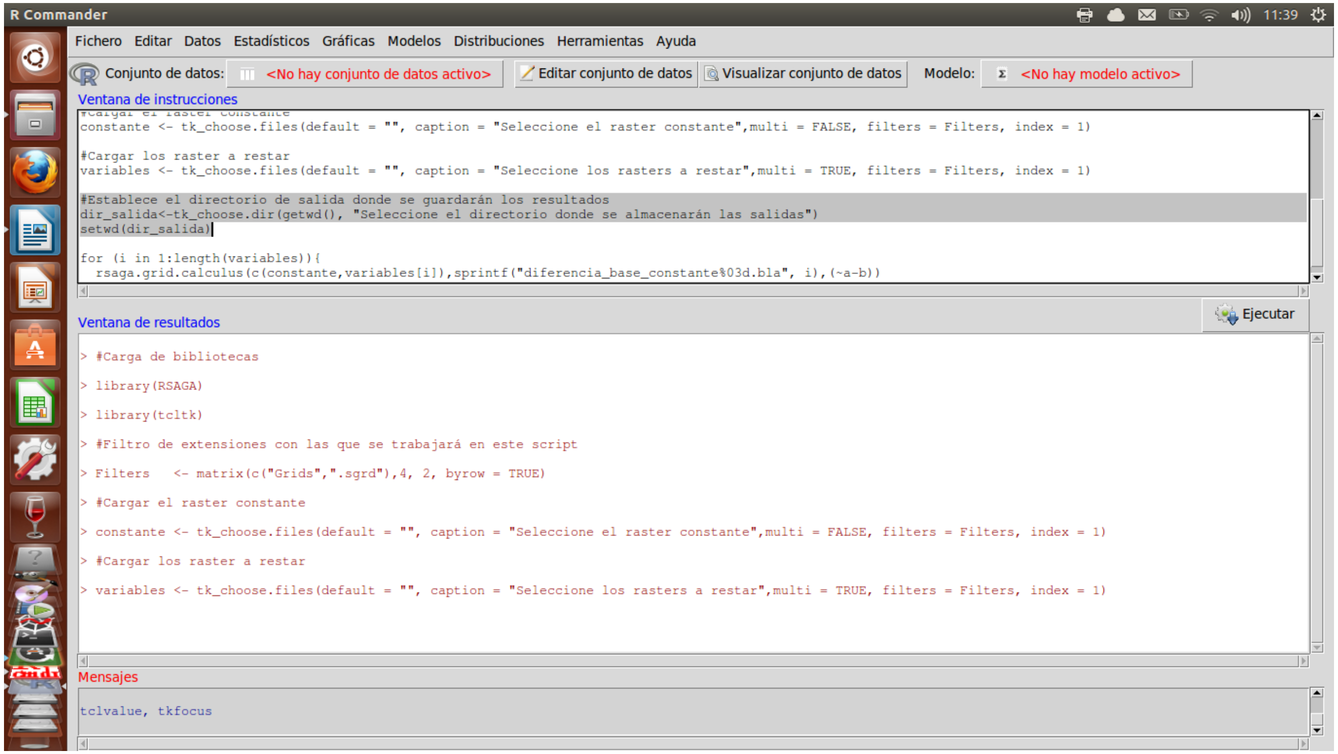

S7 MANUAL R/SAGA

The following window will open ,

- Select the folder to save the difference rasters

Usually , for the script to work out right , it is important for the output folder where the difference rasters will be saved to be in the root of the directory

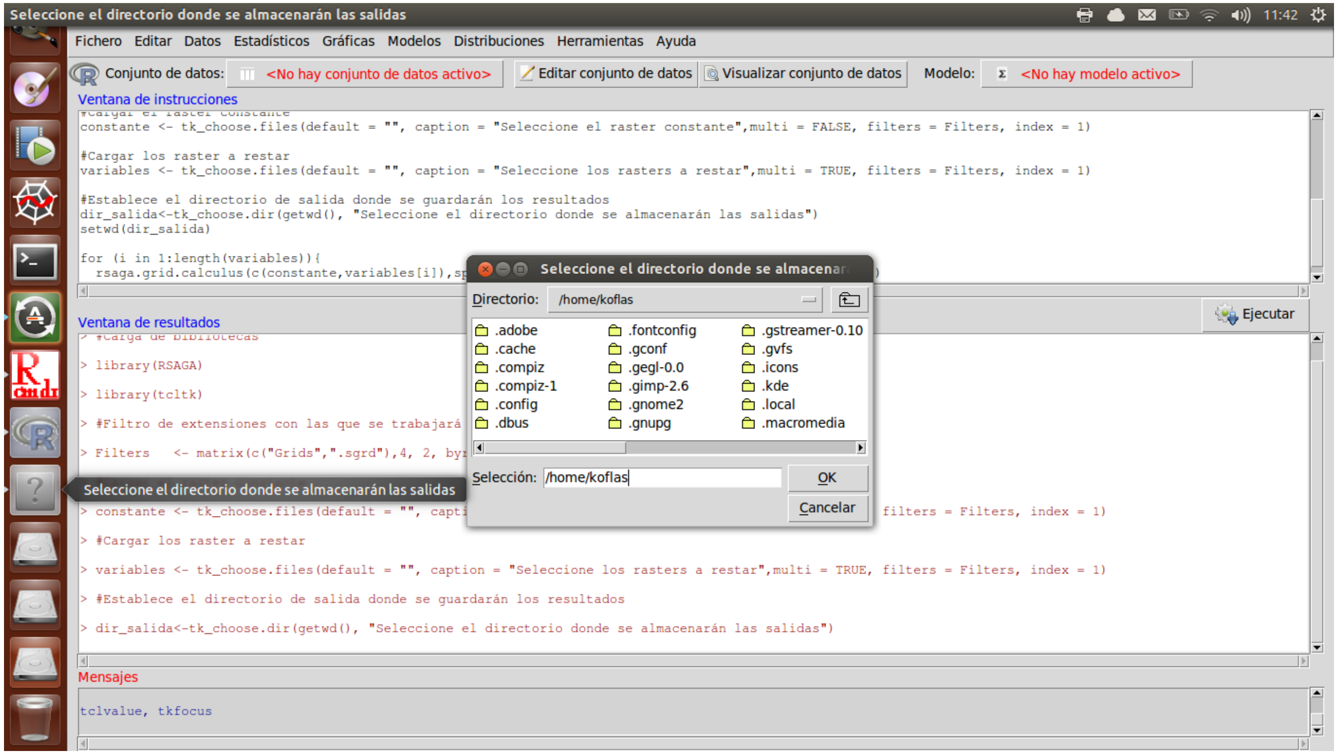

S7 MANUAL R/SAGA

- Choose the folder, the results will be expressed as .sgrd

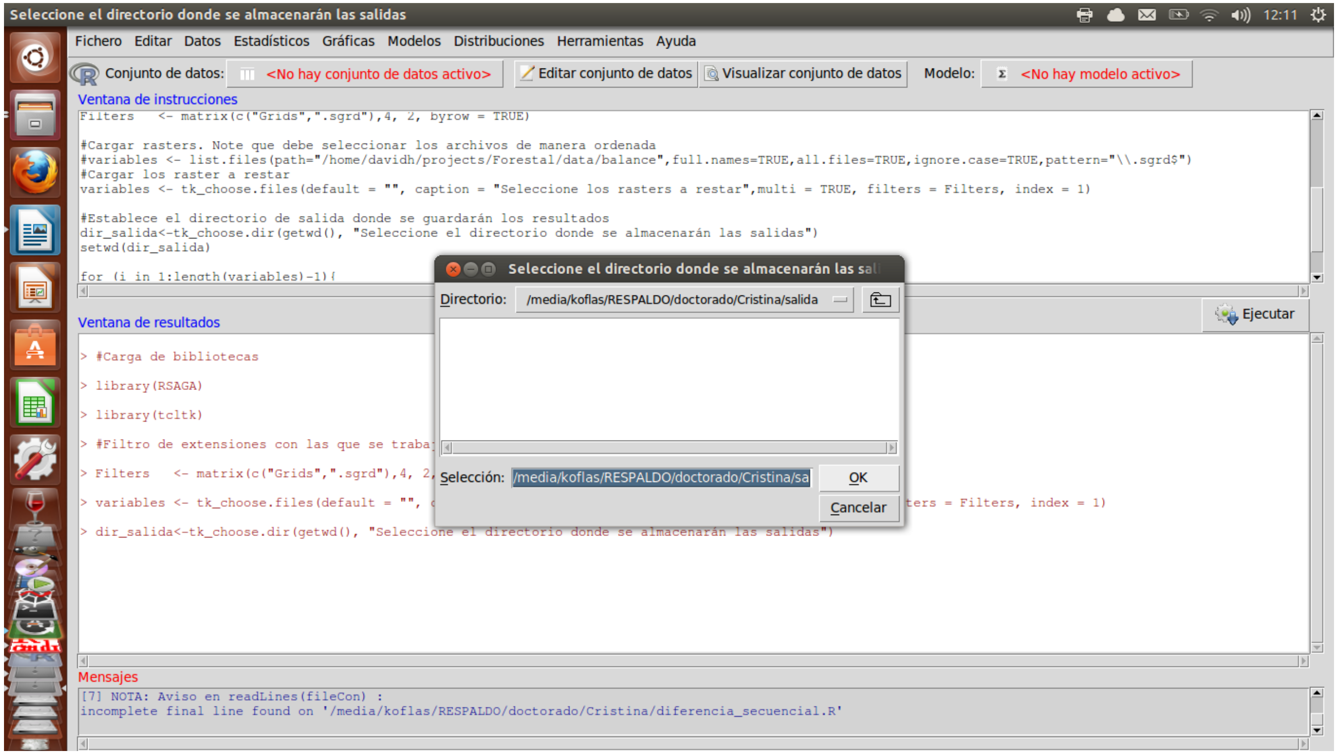

S7 MANUAL R/SAGA

- Select the code for the execution of the difference and press **Execute (Submit)**

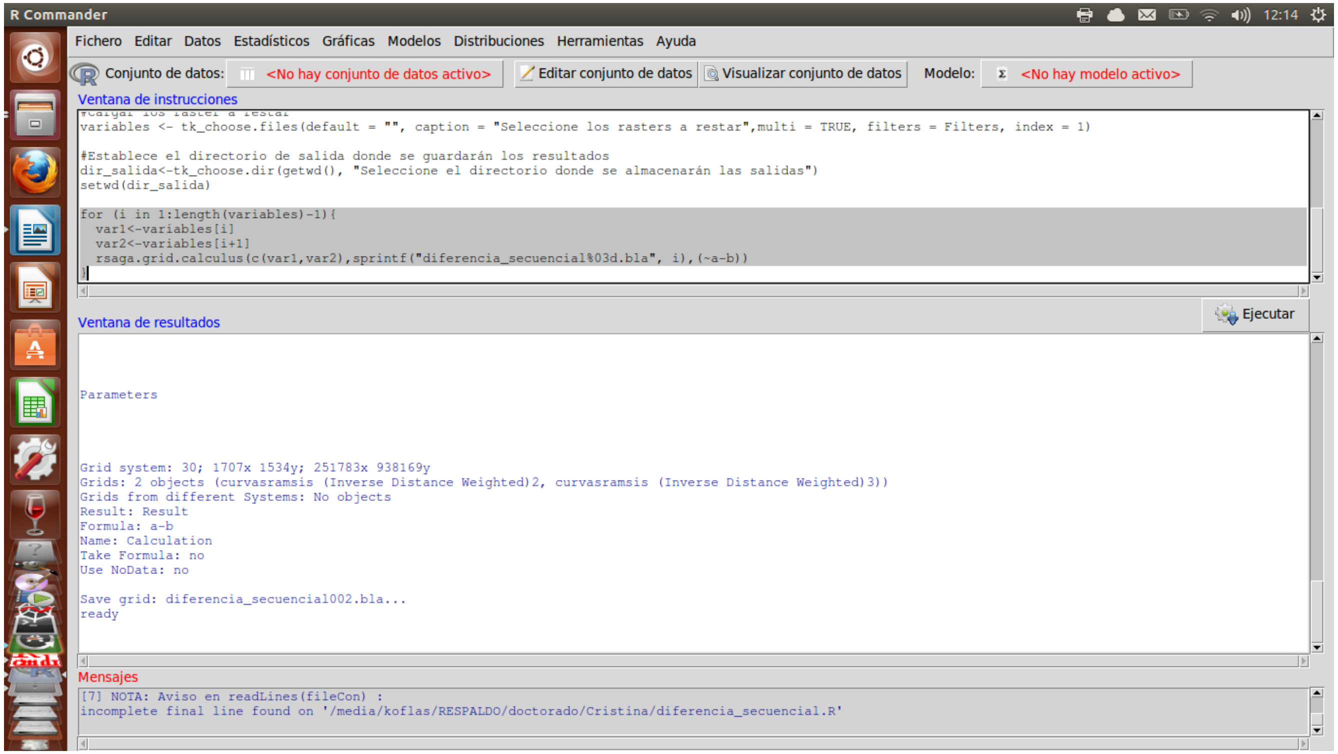

Open the output folder where the rasters were saved, note that the default name will be “diferencia_abs_secuencial” and the number of the raster. Note that a SAGA-GIS has three files **.mgrd; .sdat and .sgrd. In order to use the raster, the three files have to be in the same route, therefore, if the file is to be moved, it is better to move the folder.**

Remember that the results will be expressed as sgrd so they must be opened in SAGA-GIS

We have noticed that in the folder of the output file, in addition to the difference rasters, there will be one frame (usually the first frame used to obtain the differences which is named diferencia_secuencial000). This must be excluded from the next calculations because it is an interfering frame, not a difference raster.

S7 MANUAL R/SAGA

- Open SAGA-GIS

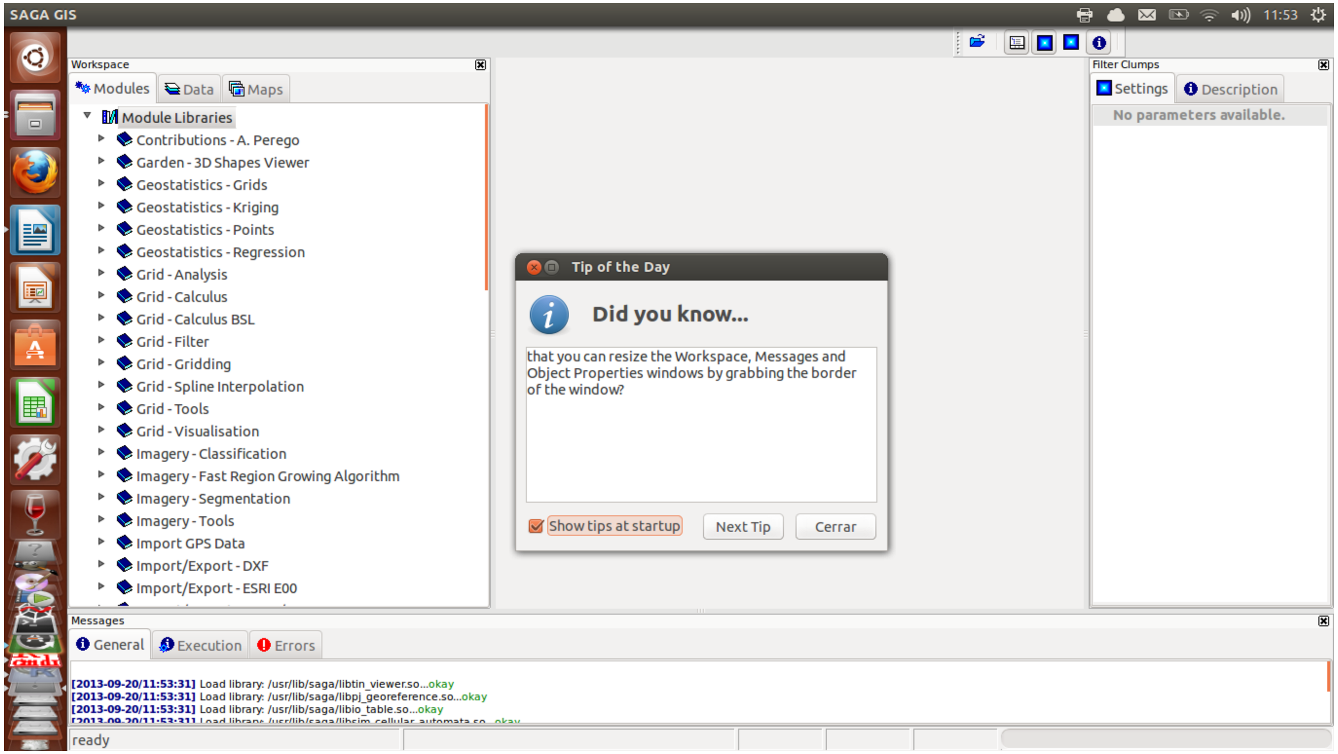

S7 MANUAL R/SAGA

- Close the window **Tip of the Day**

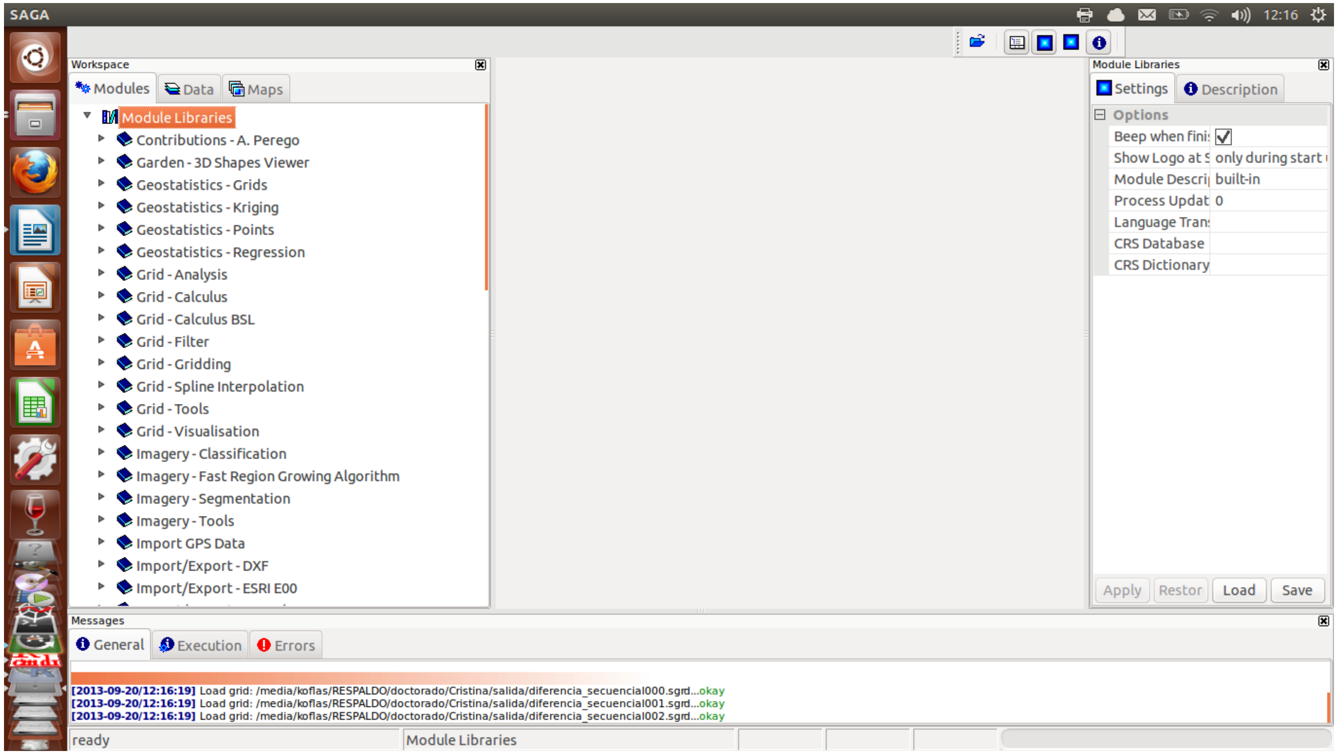

S7 MANUAL R/SAGA

Import the absolute difference rasters in the same manner as the rasters were imported at the beginning, as follows:

- Go to **Module/Import-Export GDAL/OGR**
- Go to **GDAL Import raster**
- Select the folder and the rasters that are to be imported. Remember that there will be one inappropriate frame that must be excluded from the next calculation

ADDING THE ABSOLUTE DIFFERENCE RASTERS

At this point, the absolute difference rasters are added.

- Go to **Grid Calculus**

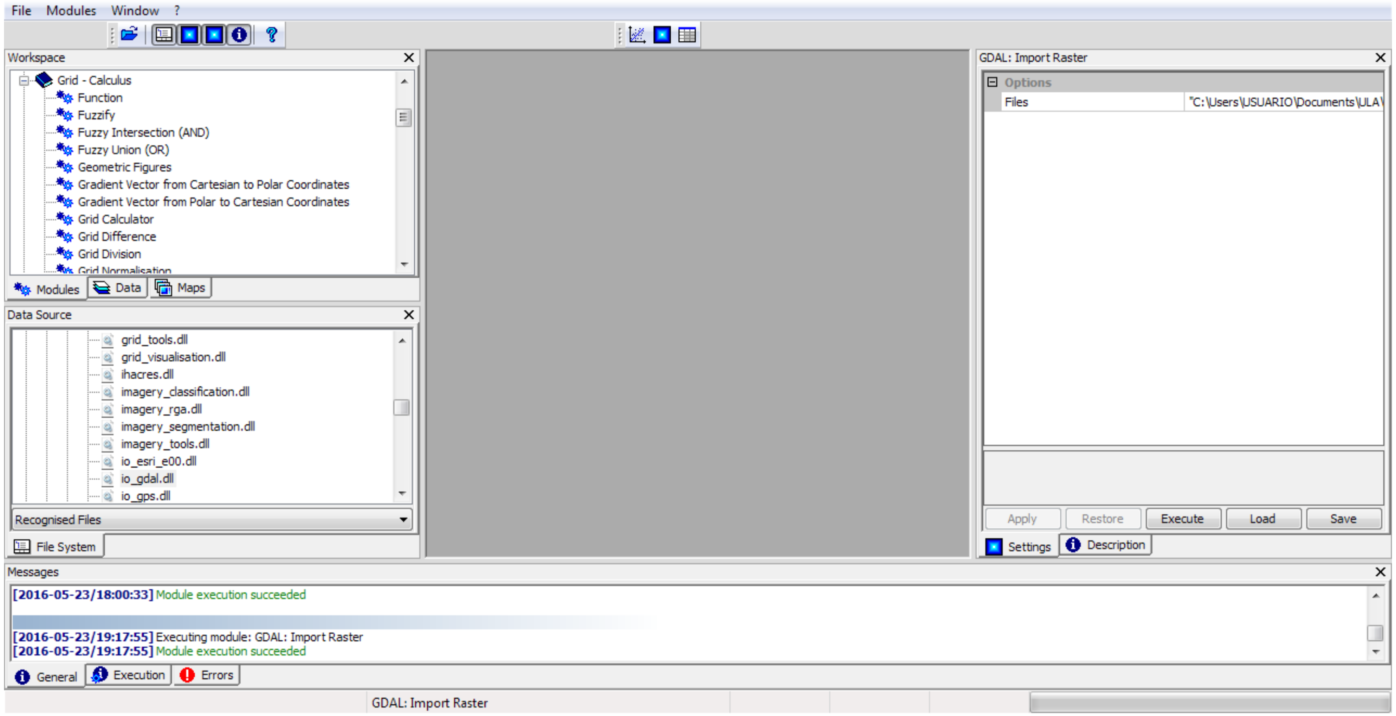

S7 MANUAL R/SAGA

- Select **Grids sum**

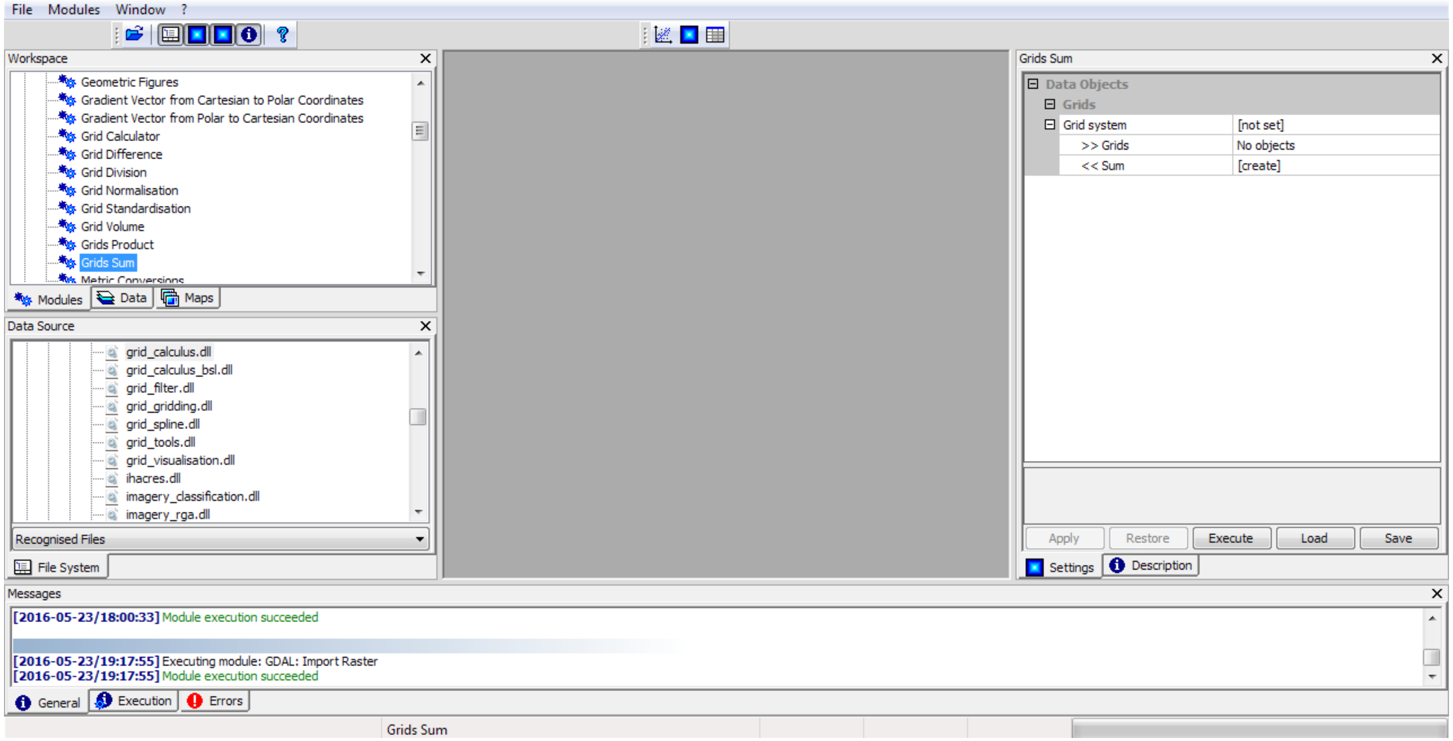

S7 MANUAL R/SAGA

- Select the rasters that are to be added

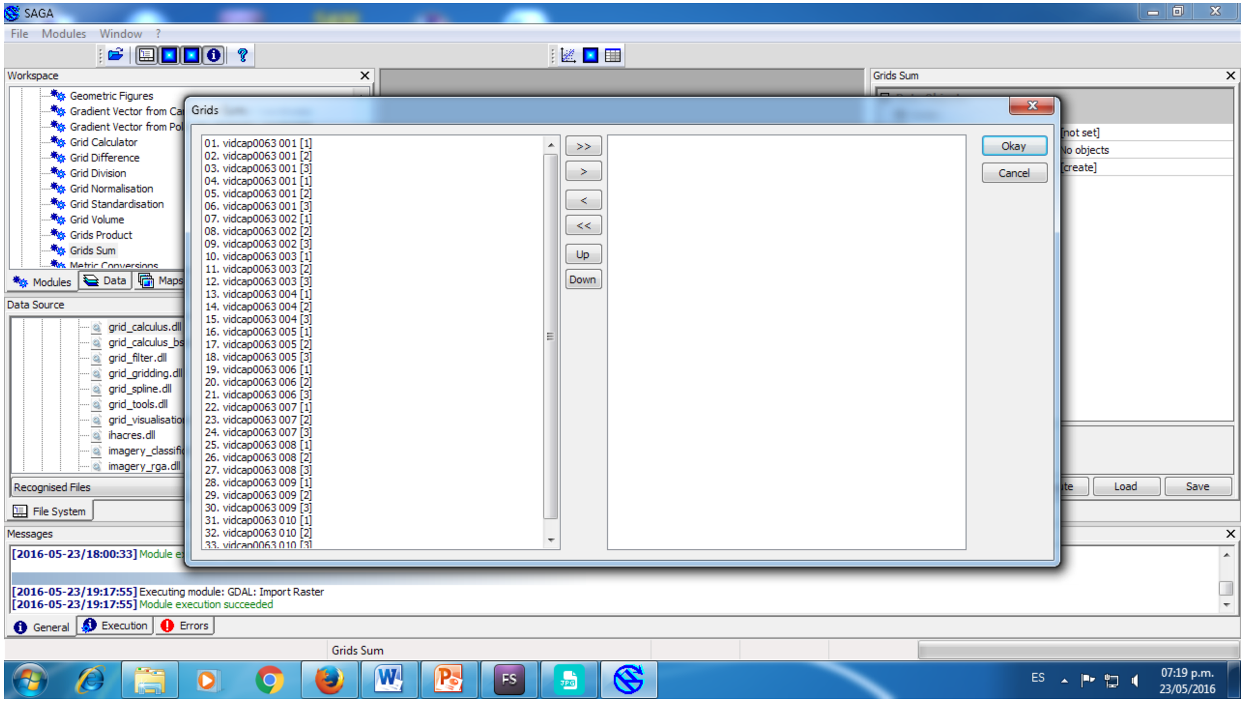

- Go to **Data**
- Click on **Sum**
- Go to **Description** on the right panel and scroll down to the **Arithmetic Mean**, which represents the **mean intensity (I)** of the sum of the absolute value of the difference rasters of a sequence of frames from a video.

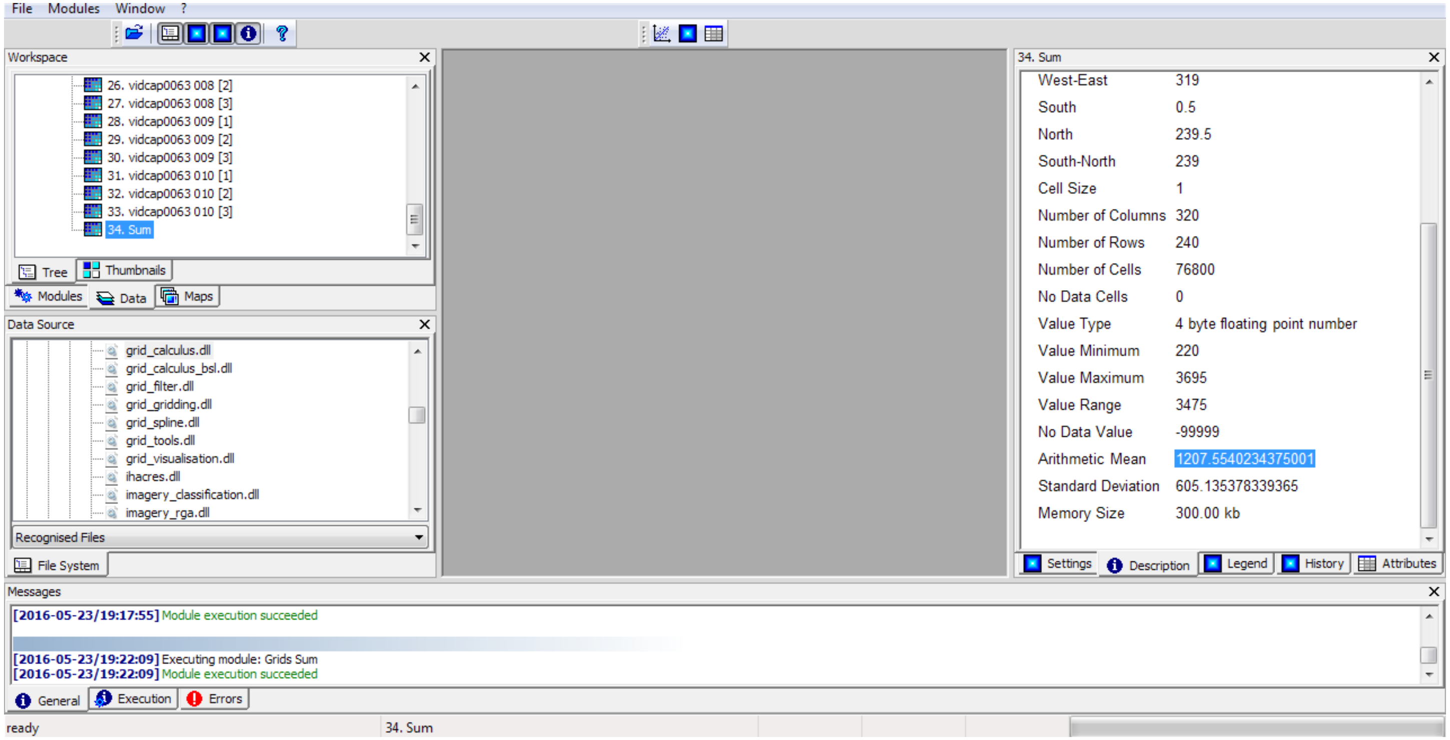

Supplement: S7 Text — User’s Manual for Script R/SAGA. (PDF) [file pntd.0005169.s008.pdf]
